# Extracellular vesicle biomarkers for pancreatic cancer diagnosis: a systematic review and meta-analysis

**DOI:** 10.1186/s12885-022-09463-x

**Published:** 2022-05-23

**Authors:** Erna Jia, Na Ren, Xianquan Shi, Rongkui Zhang, Haixin Yu, Fan Yu, Shaoyou Qin, Jinru Xue

**Affiliations:** 1 Department of Gastroenterology, The Third Hospital of Jilin University, Changchun, China; 2 Department of Thoracic Surgery, The Third Hospital of Jilin University, No. 126, Xiantai Street, Jilin Changchun, China; 3 Department of Ultrasound, Beijing Friendship Hospital of Capital Medical University, Beijing, China; 4 Department of Radiology, The First Hospital of Jilin University, Changchun, China; 5 Department of General Surgery, Union Hospital of Tongji Medical College, Huazhong University of Science and Technology, Wuhan, China

**Keywords:** Extracellular vesicle, Pancreatic cancer, Biomarker, Diagnosis

## Abstract

**Background:**

Extracellular vesicle (EV) biomarkers have promising diagnosis and screening capacity for several cancers, but the diagnostic value for pancreatic cancer (PC) is controversial. The aim of our study was to review the diagnostic performance of EV biomarkers for PC.

**Methods:**

We performed a systematic review of PubMed, Medline, and Web Of Science databases from inception to 18 Feb 2022. We identified studies reporting the diagnostic performance of EV biomarkers for PC and summarized the information of sensitivity, specificity, area under the curve (AUC), or receiver operator characteristic (ROC) curve) in according to a pre-designed data collection form. Pooled sensitivity and specificity was calculated using a random-effect model.

**Results:**

We identified 39 studies, including 2037 PC patients and 1632 noncancerous, seven of which were conducted independent validation tests. Seventeen studies emphasized on EV RNAs, sixteen on EV proteins, and sixteen on biomarker panels. MiR-10b, miR-21, and GPC1 were the most frequently reported RNA and protein for PC diagnosis. For individual RNAs and proteins, the pooled sensitivity and specificity were 79% (95% CI: 77–81%) and 87% (95% CI: 85–89%), 72% (95% CI: 69–74%) and 77% (95% CI: 74–80%), respectively. the pooled sensitivity and specificity of EV RNA combined with protein panels were 84% (95% CI: 81–86%) and 89% (95% CI: 86–91%), respectively. Surprisingly, for early stage (stage I and II) PC EV biomarkers showed excellent diagnostic performance with the sensitivity of 90% (95% CI: 87–93%) and the specificity of 94% (95% CI: 92–95%). Both in sensitivity and subgroup analyses, we did not observe notable difference in pooled sensitivity and specificity. Studies might be limited by the isolation and detection techniques of EVs to a certain extent.

**Conclusions:**

EV biomarkers showed appealing diagnostic preference for PC, especially for early stage PC. Solving the deficiency of technologies of isolation and detection EVs has important implications for application these novel noninvasive biomarkers in clinical practice.

**Supplementary Information:**

The online version contains supplementary material available at 10.1186/s12885-022-09463-x.

## Introduction

Pancreatic cancer (PC) is the seventh cancer related mortality worldwide, contributing 32, 000 deaths in 2018 [1, 2]. And its incidence and mortality is increasing in the USA and Europe over the years [2, 3]. Despite progresses in therapeutic strategies, the 5-year relative survival rate of PC still below 9%, the 5-year survival rate of patients with distant metastasis even is 2.9% [2, 4]. Surgical intervention, as the only curative strategy for patients with PC so far, is generally estimated to improve the 5-year survival rate to 30 ~ 40% for PC patients diagnosed at an early stage [5]. However, due to most of PC patients diagnosed at an advance stage, less than 20% of the tumors are eligible for surgical resection [6–8]. Imaging-based methods, such as compute tomography, magnetic resonance imaging, and echo-guided ultrasound, have been studied as screening tools only for populations at high risk of PC, which are not only expensive, radiation exposure, or less tolerant, but also have a high rate of false-positive results [9–11]. Current conventional serological biomarkers widely used for PC diagnosis and recurrence, such as carbohydrate antigen 199 (CA199), are neither sensitive nor specific enough to act as accurate early diagnostic strategies [12–16] and also are overexpressed in benign pancreatic diseases [17]. Therefore, novel circulation-based noninvasive biomarkers that can specifically and accurately diagnose PC at early stage in the general population have attracted great interest worldwide.

Extracellular vesicles (EVs), mainly classified exosomes and microvesicles, are as membrane-bound vesicles with biologically active molecules including proteins and nucleic acids and can be released from tumor cells to transport these molecules from parental cells to recipient cells to mediate tumor initiation, progression, and metastasis [18–21]. EVs stably existing in the circulation can protect these functional molecules from impaired by hydrolysis [21]. circulation EV proteins and nucleic acids beneficially classify tumor type for a diagnosis in patients with cancer of unknown primary tumor origin and the concentration of these molecules increases as cancer stage and tumor size [22–25]. Circulation EV proteins and nucleic acids as potential biomarkers for early cancers diagnosis and continuous monitoring have been investigated for a decade [26, 27]. The potential of circulation EV proteins and nucleic acids as biomarkers for PC indicates increasing application and attention. Accumulating evidences have suggested that EV proteins and nucleic acids can be as promising biomarkers for PC diagnosis [28–32]. Two recent prospective studies identified that circulation EV proteins discerned PC patients from individuals with chronic pancreatitis (CP) and healthy individuals, which showed absolute sensitivity and specificity [33, 34]. However, circulation EV biomarkers were also reported as invasive markers for benign pancreatic diseases, such as intraductal papillary mucinous neoplasms (IPMNs) [35] and CP [36, 37]. Yang et al. demonstrated that circulating EV proteins and miRNAs can predict the presence of invasive carcinoma within IPMN [35, 38]. Circulating EV miR-579-3p was identified significant lower expression in CP patients compared to healthy controls [36]. A bioinformatics analysis resulted that exsome miRNAs may be promising markers for early diagnosis and treatment of CP [39]. Therefore, the purpose of this study was to systematically review the characteristics of circulation EV biomarkers for diagnosing PC and further to evaluate the diagnosis value of these biomarkers for distinguishing PC from noncancerous.

## Methods

The systematic review and meta-analysis followed a preferred protocol and is reported according with the PRISMA guidelines [40].

### Data sources and searches

We performed an electronic search of PubMed, Medline, and Web of Science databases to identify relevant studies assessing circulation EV biomarkers for PC detection up to 18 Feb 2022. The search strategy used the following keywords combination: ([pancreatic OR pancreas] AND [cancer OR carcinoma OR neoplasm OR tumor OR malignancy OR adenocarcinoma OR adenoma] AND [detection OR diagnosis OR biomarker OR marker OR sensitivity OR specificity OR area under the curve] AND [exosome OR Extracellular Vesicles OR exosomal OR membrane vesicles OR intracellular multivesicular endosomes]). Duplicates were removed.

### Study selection

We initially screened all titles and abstracts and studies matched any of the following criteria were excluded: (1) non-English articles; (2) non-original articles; (3) not pancreatic cancer articles; (4) non-human studies; (5) not relevant to the topic. Two authors (Erna Jia and Na Ren) reviewed all potentially relevant full texts, the following studies were included: (1) studies that investigated EV biomarkers in plasma, serum, blood, or peripheral blood in PC patients; (2) PC patients were diagnosed as pancreatic ductal adenocarcinoma depending on the cytological or histological examination; (3) studies that reported the diagnostic performance of EV biomarkers for PC had relevant data (such as sensitivity, specificity, area under the curve (AUC), or receiver operator characteristic (ROC) curve); (4) control groups containing healthy people or benign disease. Any discrepancies were resolved by discussion.

### Data extraction and quality assessment

The two reviewers independently extracted available information from eligibility studies in according to a pre-designed data collection form and resolved any disagreements by discussion again. We extracted key information on first author, year of publication, country, study design, population characteristics (including sample size, mean age, and gender distribution), type of blood-based specimen, PC stage, population composition of control groups, names or panels of target biomarkers, detection methods of target biomarkers, preparation approaches of EVs, sensitivity, specificity, AUC, and *P* Value. Risk of bias and application for each study was assessed using the Quality Assessment of Diagnostic Accuracy Studies 2 (QUADAS-2) checklist [41]. Publication bias was analyzed and represented by a funnel plot, and funnel plot symmetry was assessed quantitatively with egger’s test through R software (version 3.5.3, R Foundation, Vienna, Austria) [42].

### Data synthesis and statistical analysis

We calculated mean age and sex distribution of eligibility studies using statistical software R (version 3.5.3, R Foundation, Vienna, Austria) if relevant data was not obtained but raw data was available. If the values of sensitivity, specificity or AUC were not reported, we estimated these diagnostic indicators based on ROC curve using OriginPro software (version 9.0) according to maximum Youden’s index.

We combined the sensitivity and specificity of EV biomarkers in studies reporting the relevant data to obtain a pooled diagnostic performance for PC using Meta-DiSc version 1.4 by the random-effect model (DerSimonian-Laird method). If noncancerous groups were consisted of healthy controls and/ or benign diseases, we studied them as a whole if the relevant data was reported, or we studied the healthy controls if not. We investigated heterogeneity between studies using Cocharan’s Q test and the inconsistency index (*I*^*2*^ value), with *P* < 0.05 or *I*^*2*^ > 50% as statistically significant heterogeneity. We performed sensitivity analysis for studies considered at low risk of bias and low concern for applicability to give convincing diagnostic performance of EV biomarkers for PC based on the assessment of QUADAS 2 using Review Manager 5.3. We also applied subgroup analysis to pool the sensitivity and specificity of individual EV miRNAs and individual EV RNAs detected using qPCR for PC diagnosis.

## Results

### Research results

**Fig. 1:**
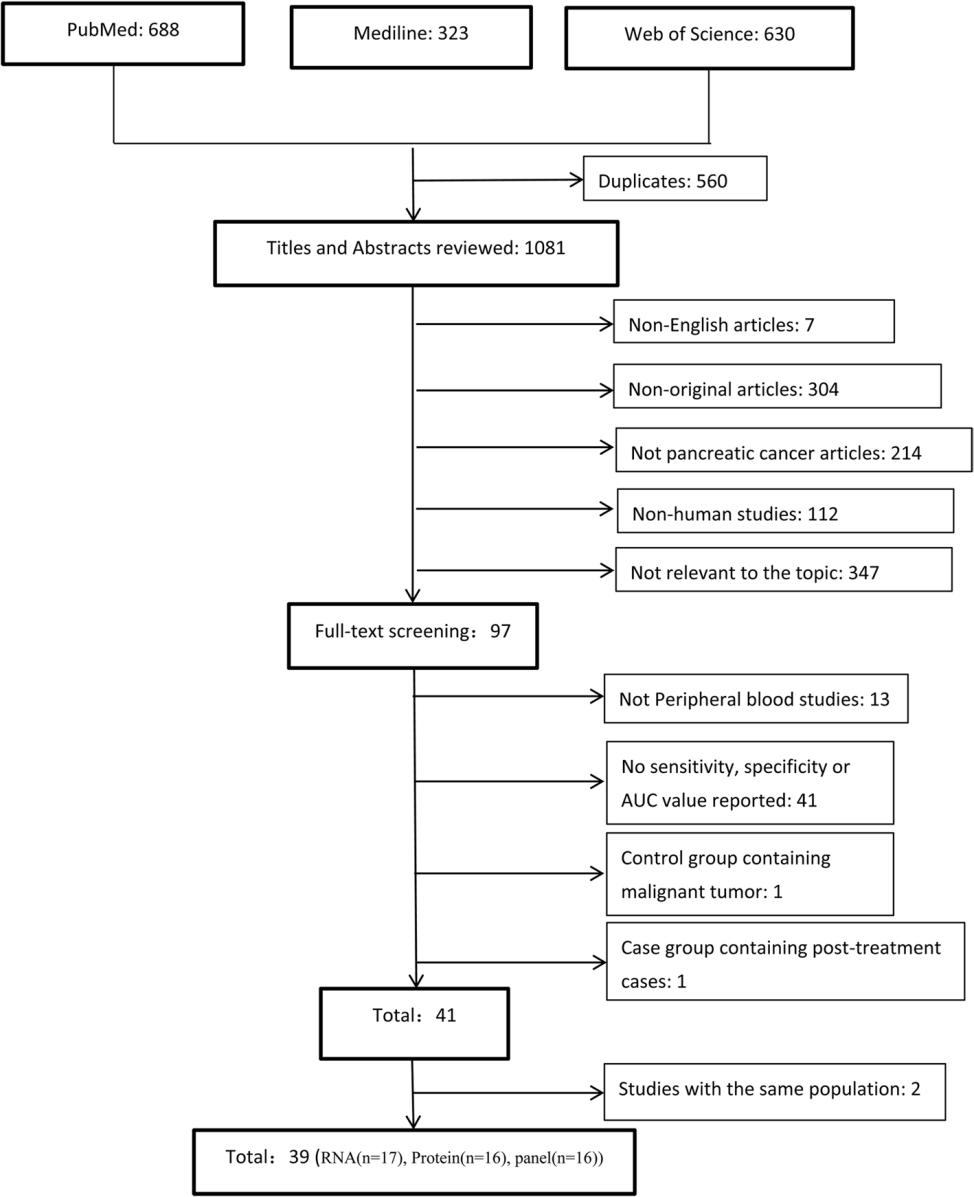
Search and selection process

We identified 1641 potentially relevant studies resulted from the above search strategy, with 688 from PubMed, 323 from Medline and 630 from Web of Science (Fig. 1). After deduplication and review of titles and abstracts, we narrowed to 97 studies for full text screening and then a further 56 studies were excluded: the specimens of 13 studies were not peripheral blood; 41 studies had no related information data, such as sensitivity, specificity, AUC value, or ROC curve; the control group of one study contained malignant tumors and the case group of one study contained post-treatment cases. Consequently, we identified 41 studies that were met the inclusion criteria [25, 30, 31, 33, 34, 43–78]. Among these 41 studies, the related information data of four studies was respectively from the same two population cohort, we selected the recently published two studies for analyses [48, 52]. Ultimately, 39 studies identifying EV biomarkers in serum or plasma for diagnosing PC were included in the systematic review [30, 31, 34, 43–57], and 34 of which were included in the meta-analysis [24, 25, 30, 31, 33, 34, 44–47, 53, 59–62, 64, 67–84].

### Study characteristics

Across 39 included studies, which involved 2037 PC cases and 1632 noncancerous, 24 studies were conducted in Asia [25, 44, 53, 60–64, 67–72, 74, 76–80, 82–85], eight were in Europe [24, 30, 31, 43, 59, 73, 75, 81], and seven were in North America [33, 34, 45–47, 58, 86]. Sample size of PC cases varied from 6 to 284 (median number, 33) and of noncancerous groups varied from 6 to 152 (median number, 29), respectively. The detail information of mean age, sex distribution, number of cases and controls, detection approaches, and PC stage in included studies were all described in Tables 1, 2 and 3. Seventeen studies focused on the diagnostic performance of individual EV RNAs (microRNAs in nine studies [25, 31, 45, 46, 59, 61, 62, 64, 68, 70, 72, 74, 78, 84], messenger RNAs in two studies [44, 53], small nucleolar RNAs in one study [53], and long noncoding RNAs in one study [80]), one of which was validated by blind external test [44], shown in Table 1; Sixteen focused on individual EV proteins (Membrane proteins (MP) in thirteen studies [24, 30, 33, 34, 47, 58, 60, 62, 75, 76, 79, 82, 83], non-membrane proteins (nMPs) in six studies [47, 58, 67, 69, 81, 82]), three of which were applied independent validation tests [30, 34, 47]. One study reported 446 individual EV proteins with AUC value greater than 0.7 and we selected 156 EV proteins with *p* value less than 0.01 for analysis [58], shown in Table 2. Sixteen studies focused on EV biomarker panels (miRNA panels in five studies [25, 31, 43, 61, 78], long RNA panels in two studies [63, 85], mRNA panels in one study [71], protein panels in six studies [33, 43, 47, 76, 79, 86], and miRNA combined with protein panels in four study [43, 62, 73, 77]), twelve panels in six studies were conducted independent validation tests [43, 47, 63, 71, 78, 85], one study reported 129 miRNA panels and we selected 119 panels with AUC value greater than 0.70 for analysis [78], shown in Table 3. Nine studies reported the diagnostic performance of EV biomarkers for early stage (stage I and II) PC [30, 34, 46, 53, 60, 75, 84–86].

**Table 1:** Diagnostic performance of RNAs in extracellular vesicles for pancreatic cancer

Study	Country	study design	Cases vs Controls			Specimen	Stage	Status Controls	Detection Method	markers	SEN%	SPE%	AUC	P Value
			**Number**	**Age**	**Male (%)**									
										**miRNA**				
Xu, 2017 [46]	USA	Case–control	15/15	67/48	53/27	Plasma	I-IIA	HC	qPCR	miR-196a	87^e^	73^e^	0.81	< 0.001
										miR-1246	67^e^	80^e^	0.73	0.019
										miR-196b	67^e^	80^e^	0.71	0.033
Lai, 2017 [45]	USA	Case–control	29/6	67/NA	52/NA	Plasma	I-IV	HC	qPCR	miR-10b	100	100	1.00	< 0.001
										miR-21	100	100	1.00	< 0.001
										miR-30c	100	100	1.00	< 0.001
										miR-106b	62	100	0.85	0.007*
										miR-20a	83	100	0.95	< 0.001
										miR-181a	100	100	1.00	< 0.001
										miR-let7a	100	100	1.00	< 0.001
										miR-122	93	100	0.99	< 0.001
Goto, 2018 [84]	Japan	Case–control	32/22	64/58	53/64	Serum	I-IV	HC	qPCR	miR-191	72	84	0.79	0.001
										miR-21	81	81	0.83	< 0.001
										miR-451a	66	86	0.76	0.002
			9/22	NA/58	NA/64	Serum	I-IIA	HC	qPCR	miR-191	67	84	0.75	0.032
										miR-21	67	81	0.74	0.004
										miR-451a	67	86	0.74	0.044
			23/22	NA/58	NA/64	Serum	IIB-IV	HC	qPCR	miR-191	79	79	0.80	0.001
										miR-21	86	81	0.86	< 0.001
										miR-451a	70	81	0.77	0.002
Zhou, 2020 [62]	China	Case–control	30/10	60/58	/	Plasma	I-IV	NC^b^	3D mircrofluidic chip	miR-451a	81^e^	100^e^	0.93	/
										miR-21	87^e^	100^e^	0.94	/
										miR-10b	79^e^	99^e^	0.88	/
										**miRNA**				
Reese, 2020 [31]	Germany	Case–control	56/22	NA/68	64/50	Serum	II-IV	HC	qPCR	miR-200b	67^e^	76^e^	0.79	0.0001
									qPCR	miR-200c	51^e^	90^e^	0.67	0.0239
			56/11	NA/62	64/55	Serum	II-IV	CP	qPCR	miR-200b	63^e^	86^e^	0.77	0.0047
			56/33		64/52	Serum	II-IV	NC^c^	qPCR	miR-200b	85^e^	63^e^	0.77	0.005
			56/22	NA/68	64/50		II-IV	HC	qPCR	miR-200b	63^e^	68^e^	0.69	0.0077
Wu, 2020 [61]	China	Case–control	30/10	62/51	60/80	serum	0-IV	CP	qPCR	miR-21	80	90	0.87	/
										miR-210	83	90	0.82	/
Pu, 2020 [25]	China	Case–control	36/65	/	/	Plasma	I-IV	HC	cationic lipoplex nanoparticle	miR-21	53^e^	98^e^	0.72	0.0003
										miR-10b	42^e^	100^e^	0.65	0.0105
Flammang, 2020 [59]^a^	Germany	Case–control	44/12	/	/	serum	II-IV	HC	qPCR	miR-192-5p	64^e^	99^e^	0.83	0.0004
Wang, 2021 [64]	China	Case–control	17/12	/	/	serum	/	PBT	qPCR	miRNA-1226-3p	75^e^	66^e^	0.74 /	
Xiao, 2021 [70]	China	Case–control	10/10	/	/	Plasma		HC	PNA-functionalized nanochannel sensor	miR-10b	/	/	0.99	/
Shao, 2021 [74]	China	Case–control	63/22	60/50	56/41	serum	I-IV	HC	qPCR	miR-483-3p	82^e^	56^e^	0.69	/
Wang L, 2021 [72]	China	Case–control	62/53	/	/	plasma		HC	qPCR	miR-19b-3p	85	91	0.94	< 0.001
			62/23	/	/			CP			81	87	0.90	< 0.001
			62/30	/	/			OPT			94	63	0.81	< 0.001
Chen, 2022 [68]	China	Case–control	191/90	62/57	57/52	serum	I-IV		HC PBD	miR-451a	80	87	0.90	/
			191/95	62/59	57/58						71	89	0.86	/
										**LncRNA**				
Takahashi, 2019 [80]	Japan	Case–control	20/43	72/71	40/42	Serum	II-IV	NC^d^	dPCR	HULC	80	92	0.92	/
			20/21	72/72	40/36	Serum	II-IV	HC	dPCR	HULC	80	95	0.94	/
			20/22	72/70	40/48	Serum	II-IV	IPMN	dPCR	HULC	85	83	0.91	/
Guo, 2021 [78]^a^	China	Case–control	27/15	58/44	63/60	plasma	IB-IV	CP	small RNA sequencing	miR-95-3p	92^e^	94^e^	0.91	
										miR-199b-3p	/	/	0.90	
										miR-3158-3p	/	/	0.90	
										miR-199a-3p	/	/	0.89	
										miR-4732-3p	/	/	0.88	
										miR-10a-5p	/	/	0.88	
										miR-145-3p	/	/	0.87	
										miR-27b-3p	/	/	0.86	
										miR-511-5p	/	/	0.86	
										miR-7706	/	/	0.85	
										miR-99b-5p	/	/	0.85	
										miR-143-3p	/	/	0.83	
										miR-486-3p	/	/	0.83	
										miR-99a-5p	/	/	0.83	
										miR-223-3p	/	/	0.82	
										**mRNA**				
Hu, 2017 [44]^a^	China	Case-control^d^	20/15	/	/	Serum	I-IV	HC	LPHN-CHDC biochip	GPC1 mRNA	**95**	**93**	**0.94**	/
										**snoRNA**				
Kitagawa, 2019 [53]^a^	Japan	Case–control	27/13	/	63/31	Serum	I-III	BGD	qPCR	SNORA14B	93^e^	69^e^	0.88	/
										SNORA18	81^e^	84^e^	0.88	/
										SNORA25	92^e^	76^e^	0.90	/
										SNORA74A	92^e^	84^e^	0.91	/
										SNORD22	70^e^	93^e^	0.86	/
										**snoRNA**				
Kitagawa, 2019 [53]	Japan	Case–control	27/13	/	63/31	Serum	I-III	BGD	qPCR	CCDC88A	55^e^	92^e^	0.72	/
										ARF6	85^e^	92^e^	0.94	/
										Vav3	74^e^	85^e^	0.84	/
										WASF2	93^e^	84^e^	0.94	/
			8/13	/	NA/31	Serum	I-IIA	BGD	qPCR	SNORA14B	/	/	0.86	/
										SNORA18	/	/	0.91	/
										SNORA25	100^e^	78^e^	0.91	/
										SNORA74A	100^e^	84^e^	0.95	/
										SNORD22	/	/	0.97	/
										CCDC88A	/	/	0.73	/
										ARF6	100^e^	92^e^	0.98	/
										Vav3	/	/	0.89	/
										WASF2	100^e^	84^e^	0.97	/
			19/13	/	NA/31	Serum	IIB-III	BGD	qPCR	SNORA14B	/	/	0.88	/
										SNORA18	/	/	0.88	/
										SNORA25	93^e^	77^e^	0.90	/
										SNORA74A	93^e^	85^e^	0.91	/
										SNORD22	/	/	0.86	/
										CCDC88A	/	/	0.72	/
										ARF6	85^e^	92^e^	0.94	/
										Vav3	/	/	0.84	/
										WASF2	93^e^	84^e^	0.94	/

SENs, SPEs and AUCs in bold fonts represent results from validation set (non-bold fonts represent results without validation)

*AUC* area under the curve, *BGD* benign gastrointestinal disease, *CP* chronic pancreatitis, *HC* healthy control, *IPMN* intraductal papillary mucinous neoplasm, *LPHN-CHDC* lipid polymer hybrid nanoparticle-catalyzed hairpin DNA circuit, *NC* noncancerous, *PBT* pancretian benign tumor, *SEN* sensitivity, *SPE* specificity, *OPT* other pancreatic tumor (pancreatic neuroendocrine tumor, solid pseudopapillary tumor, serous or mucinous cystadenomas, intraductal papillary mucinous neoplasms, and epithelial cysts), *PBD* pancreatic benign disease, *PNA* peptide nucleic acid, *OPT* pancreatic neuroendocrine tumor, solid pseudopapillary tumor, serous or mucinous cystadenomas, intraductal papillary mucinous neoplasms, and epithelial cysts

^a^represent markers extracted from extracellular vesicles

^b^no history of cancer

^c^HC and CP

^d^HC and IPMN

^e^represent estimated value

*p* value indicates *p* value of AUC

**Table 2:** Diagnostic performance of proteins in extracellular vesicles for pancreatic cancer

Study	Country	study design	Cases vs Controls			Specimen	Stage	Status Controls	Detection Method	markers	SEN%	SPE%	AUC	*P* Value
			**Number**	**Age**	**Male (%)**									
										**MPs**				
Melo, 2015 [30]	Germany	Case–control	5/126	/	/	Serum	0-I	NC^b^	Flow cytometry	GPC1	100	100	1.00	/
			18/126	/	/	Serum	IIa		Flow cytometry	GPC1	100	100	1.00	/
			117/126	/	/	Serum	IIb		Flow cytometry	GPC1	100	100	1.00	/
			11/126	/	/	Serum	III		Flow cytometry	GPC1	100	100	1.00	/
			41/126	/	/	Serum	IV		Flow cytometry	GPC1	100	100	1.00	/
		Case–control	56/26	70/NA	50/NA	Serum	I-IV		Flow cytometry	GPC1	**100**	**100**	**1.00**	/
Liang, 2017 [34] ^a^	USA	Case–control	49/48	NA/62	NA/40	Plasma	I-III	HC	nPES assay	EphA2	**94**	**85**	**0.96**	< 0.001
			49/48	NA/52	NA/52	Plasma	I-III	CP	nPES assay	EphA2	**89**	**85**	**0.94**	< 0.001
			37/48	67/62	60/40	Plasma	I-II	HC	nPES assay	EphA2	**91**	**85**	**0.96**	< 0.001
			37/48	67/52	60/52	Plasma	I-II	CP	nPES assay	EphA2	**86**	**85**	**0.93**	< 0.001
Yang, 2017 [47]^a^	USA	Prospective	22/21	/	/	plasma	/	NC^c^	NPS chip	GPC1	**82**	**52**	/	/
										EGFR	**59**	**76**	/	/
										EPCAM	**45**	**95**	/	/
										HER2	**59**	**85**	/	/
										MUC1	**36**	**90**	/	/
Li, 2018 [82]	China	Case–control	34/32	NA/50	NA/69	Serum	/	HC	SERS immunosensor	GPC1	59	58	0.67	/
			34/32	NA/50	NA/69	Serum	/	HC	SERS immunosensor	EGFR	57	56	0.63	/
Jin, 2018 [49, 83]	China	Case–control	24/46	61/49	33/50	Serum	I-IV	HC	ELISA	ZIP4	92	80	0.89	/
			24/32	61/44	33/28	Serum	I-IV	NC^d^	ELISA	ZIP4	92	84	0.89	/
			24/32	61/63	33/53	Serum	I-IV	BD	ELISA	ZIP4	92	81	0.81	/
Xiao, 2019 [79]	China	Case–control	24/26	59/45	42/85	Plasma	/	HC	Flow cytometry	GPC1	72*	84*	0.89	/
										CD82	86*	66*	0.85	/
			24/6	59/73	42/83	Plasma	/	CP	Flow cytometry	GPC1	100*	66*	0.85	/
										CD82	100*	66*	0.90	/
Buscail, 2019 [24, 54, 66]	France	Prospective	22/28	70/58	59/28	Serum-CD63	I-III	NC^e^	Flow cytometry	GPC1	50	90	0.68	/
										**MPs**				
Rodrigues, 2019 [33] ^a^	USA	Case–control	20/12	/	45/25	Serum	/	NC^k^	nanoparticle-and dyebased fluorescent immunoassay	EpCAM	75*	100*	0.92	
										EphA2	95*	83*	0.93	
Zhou, 2020 [62]	China	Case–control	30/10	60/58	/	Plasma	I-IV	NC^f^	3D mircrofluidic chip	EphA2	69*	99*	0.85	
Wei, 2020 [60]	China	Case–control	204/74	62/NA	/	serum	I-IV	HC	Enzyme-Linked Immunosorbent Assays	EphA2	83	94	0.94	
			204/75	62/NA	/			BPD		EphA2	81	94	0.92	
			94/74	/	/		I-II	HC		EphA2	83	94	0.92	
			94/75	/	/			BPD		EphA2	84	88	0.90	
Fahrmann,2020 [58]^a^	USA	Case–control	6/21	/	/	Plasma	IV	HC	SOMAscan assay	ASGR1	/	/	0.89	0.0026
										ANXA1	/	/	0.99	0.0000
										IL12RB2	/	/	0.85	0.0083
										ADAM9	/	/	0.98	0.0000
										TGFBR2	/	/	0.85	0.0083
										EDA	/	/	0.85	0.0083
										BMPR2	/	/	0.88	0.0034
										TYRO3	/	/	0.87	0.0043
										FLRT3	/	/	0.87	0.0054
										SLAMF6	/	/	0.95	0.0002
										CD4	/	/	0.87	0.0054
										RXFP1	/	/	0.87	0.0054
Li, 2021 [76] ^a^	China	Case–control	21/29	54/62	43/55	plasma	/	HC	AbMB-bioChol paltform	EGFR	52*	93*	0.74	
										EpCAM	94*	48*	0.68	
										GPC1	43*	89*	0.72	
										EphA2	47*	86*	0.64	
										**MPs**				
Moutinho-R, 2021 [75]	Portugal	prospective	60/29	67/54	55/76	serum	I-IV	CP	flow cytometry	GPC1	98	86	0.96	< 0.0001
			15/29	NA/54	NA /76		I				100*	96*	0.97	
			45/29	NA /54	NA /76		II-IV				95*	97*	0.96	
										**nMPs**				
Yang, 2017 [47] ^a^	USA	Prospective	22/21	/	/	plasma	/	NC^c^	NPS chip	WNT2	**64**	**76**	/	/
										GRP94	**55**	**71**	/	/
		Case–control	22/10	68/48	36/40	plasma	II-IV	HC	NPS chip	B7-H3	50	100	0.75	/
Li, 2018 [82]	China	Case–control	71/32	60/50	54/69	Serum	I-IV	HC	SERS immunosensor	MIF	63	76	0.89	/
Lux, 2019 [54, 81]	Germany	Case–control	55/34	67/NA	53/NA	Serum	I-IV	NC^g^	Flow cytometry	c-Met	70	85	0.75*	/
Fahrmann,2020 [58]^a^	USA	Case–control	6/21	/	/	Plasma	IV	HC	SOMAscan assay	IFNGR2	/	/	0.87	0.0054
										TPSG1	/	/	0.94	0.0003
Yang, 2021 [69]	China	Case–control	62/42	/	48/ NA	plasma	I-IV	NC^h^	Enzyme-linked immunosorbert assay	ALIX	53	84	0.73	0.0003
Zheng, 2022 [67]	China	Case–control	57/84	/	31/39	plasma	II-IV	HC	MALDI-TOF MS	SAA1	96	28	0.61	/
										FGG	40	87	0.62	/
												**nMPs**		
Fahrmann,2020 [58]^a^	USA	Case–control	6/21	/	/	Plasma	IV	HC	SOMAscan assay	MAPKAPK2	/	/	0.94	0.0004
										PDXK	/	/	0.94	0.0003
										PLCG1	/	/	0.94	0.0004
										INSR	/	/	0.94	0.0004
										IL34	/	/	0.93	0.0006
										AIP	/	/	0.93	0.0006
										YES1	/	/	0.93	0.0006
										DYNLRB1	/	/	0.93	0.0006
										IDE	/	/	0.93	0.0006
										CCL28	/	/	0.93	0.0006
										**nMPs**				
Fahrmann,2020 [58]^a^	USA	Case–control	6/21	/	/	Plasma	IV	HC	SOMAscan assay	C5 C6	/	/	0.92	0.0008
										CD109	/	/	0.92	0.0008
										CAMK2A	/	/	0.92	0.0008
										TNFSF14	/	/	0.91	0.0011
										MMP14	/	/	0.91	0.0011
										PRKCI	/	/	0.91	0.0011
										MAP2K1	/	/	0.91	0.0011
										KYNU	/	/	0.91	0.0011
										KIF23	/	/	0.91	0.0011
										IL27RA	/	/	0.91	0.0011
										IMPDH1	/	/	0.91	0.0011
										TNFRSF13B	/	/	0.91	0.0011
										SEMA6B	/	/	0.91	0.0011
										AIMP1	/	/	0.91	0.0011
										NMT1	/	/	0.90	0.0015
										EPHA5	/	/	0.90	0.0015
										MSLN	/	/	0.90	0.0015
										IBSP	/	/	0.90	0.0015
										TNFSF13B	/	/	0.90	0.0015
										CTF1	/	/	0.90	0.0015
										BCL2L1	/	/	0.90	0.0015
										IL1A	/	/	0.90	0.0015
										MAPK12	/	/	0.90	0.0015
										FABP1	/	/	0.90	0.0020
										CCL17	/	/	0.90	0.0020
										**nMPs**				
Fahrmann,2020 [58]^a^	USA	Case–control	6/21	/	/	Plasma	IV	HC	SOMAscan assay	SPARCL1	/	/	0.90	0.0020
										LCK	/	/	0.90	0.0020
										CCL3L1	/	/	0.90	0.0020
										PDE9A	/	/	0.90	0.0020
										PRDX5	/	/	0.90	0.0020
										CD300C	/	/	0.90	0.0020
										BCL2L2	/	/	0.90	0.0020
										CSRP3	/	/	0.90	0.0020
										GDF2	/	/	0.90	0.0020
										IL17RB	/	/	0.90	0.0020
										LGALS3BP	/	/	0.90	0.0020
										SEMA6A	/	/	0.90	0.0020
										CLEC11A	/	/	0.90	0.0020
										UBC	/	/	0.89	0.0026
										HIPK3	/	/	0.89	0.0026
										PARK7	/	/	0.89	0.0026
										MMP13	/	/	0.89	0.0026
										FGFR4	/	/	0.89	0.0026
										FGF5	/	/	0.89	0.0026
										DDR1	/	/	0.89	0.0026
										PDK1	/	/	0.89	0.0026
										CXCL8	/	/	0.89	0.0026
										GDF11	/	/	0.89	0.0026
										IDUA	/	/	0.89	0.0026
										CA3	/	/	0.89	0.0026
										**nMPs**				
Fahrmann,2020 [58]^a^	USA	Case–control	6/21	/	/	Plasma	IV	HC	SOMAscan assay	DYNLL1	/	/	0.89	0.0026
										ACVRL1	/	/	0.89	0.0026
										CXCL1	/	/	0.88	0.0034
										CRLF1/CLCF1	/	/	0.88	0.0034
										DHH	/	/	0.88	0.0034
										ENTPD5	/	/	0.88	0.0034
										UBE2G2	/	/	0.88	0.0034
										IFNG	/	/	0.88	0.0034
										CHEK2	/	/	0.88	0.0034
										LTA LTB	/	/	0.87	0.0043
										FAM107A	/	/	0.87	0.0043
										TNFRSF18	/	/	0.87	0.0043
										KLK6	/	/	0.87	0.0043
										PPP3R1	/	/	0.87	0.0043
										CD244	/	/	1.00	0.0000
										ETHE1	/	/	0.87	0.0043
										PDXP	/	/	0.87	0.0043
										IFNB1	/	/	0.87	0.0043
										IL36A	/	/	0.87	0.0043
										KLK14	/	/	0.87	0.0043
										IL1RN	/	/	0.87	0.0043
										F3	/	/	0.87	0.0043
										MAPK11	/	/	0.87	0.0054
										TNFRSF14	/	/	0.87	0.0054
										PRLR	/	/	0.87	0.0054
										**nMPs**				
Fahrmann,2020 [58]^a^	USA	Case–control	6/21	/	/	Plasma	IV	HC	SOMAscan assay	VEGFA	/	/	0.87	0.0054
										FABP5	/	/	0.87	0.0054
										CDH3	/	/	0.87	0.0054
										ISG15	/	/	0.87	0.0054
										CKM	/	/	0.87	0.0054
										ABL2	/	/	0.87	0.0054
										TNFSF11	/	/	0.86	0.0067
										CA10	/	/	0.86	0.0067
										EPHA2	/	/	0.86	0.0067
										HK2	/	/	0.86	0.0067
										STK16	/	/	0.86	0.0067
										CD47	/	/	0.86	0.0067
										PAK7	/	/	0.86	0.0067
										CDK2 CCNA2	/	/	0.86	0.0067
										ZAP70	/	/	0.86	0.0067
										CXCL5	/	/	0.86	0.0067
										MAPK8	/	/	0.86	0.0067
										IL17F	/	/	0.86	0.0067
										PDGFRA	/	/	0.86	0.0067
										FGF2	/	/	0.86	0.0067
										AFP	/	/	0.86	0.0067
										PECAM1	/	/	0.86	0.0067
										STAT6	/	/	0.85	0.0083
										TOP1	/	/	0.85	0.0083
										CRLF2	/	/	0.85	0.0083
										**nMPs**				
Fahrmann,2020 [58]^a^	USA	Case–control	6/21	/	/	Plasma	IV	HC	SOMAscan assay	TLR4	/	/	0.85	0.0083
										BCL2	/	/	0.85	0.0083
										TNF	/	/	0.85	0.0083
										C1QBP	/	/	0.85	0.0083
										CLEC11A	/	/	0.85	0.0083
										GDF9	/	/	0.85	0.0083
										ING1	/	/	0.85	0.0083
										BIRC5	/	/	0.85	0.0083
										B2M	/	/	0.85	0.0083
										ERP29	/	/	0.85	0.0083
										FLT3LG	/	/	0.85	0.0083
										NAPA	/	/	0.85	0.0083
										GCKR	/	/	0.85	0.0083
										EPHA1	/	/	0.85	0.0083
										HSD17B10	/	/	0.85	0.0083
										CEBPB	/	/	0.85	0.0083
										SSRP1	/	/	0.85	0.0083
										SPHK2	/	/	0.85	0.0083
										CCL22	/	/	0.85	0.0083
										GDF5	/	/	0.85	0.0083
										APOD	/	/	0.85	0.0083
										POR	/	/	0.99	0.0000
										LY9	/	/	0.97	0.0001
										IFNGR1	/	/	0.96	0.0001
										GSK3A/B	/	/	0.96	0.0001
										**nMPs**				
Fahrmann,2020 [58]^a^	USA	Case–control	6/21	/	/	Plasma	IV	HC	SOMAscan assay	FLT3	/	/	0.95	0.0002
										CSNK2A1	/	/	0.95	0.0002
										PIK3CA/R1	/	/	0.95	0.0002
										LIN7B	/	/	0.95	0.0002
										METAP1	/	/	0.95	0.0002
										MAPK13	/	/	0.90	0.0015
										FAS	/	/	0.94	0.0003

SENs, SPEs and AUCs in bold fonts represent results from validation set (non-bold fonts represent results without validation)

*AUC* area under the curve, *BD* Biliary disease, *BPD* benign pancreatic disease, *CP* chronic pancreatitis, *HC* healthy control, *IPMN* intraductal papillary mucinous neoplasm, *NC* noncancerous, *nPES* nanoplasmon-enhanced scattering, *NPS* nanoplasmonic sensor, *SERS* surface-Enhanced Raman Scattering, *SOMAscan assay* slow off-rate modified DNA aptamer, *nMPs* nonMembrane proteints, *MPs* Membrane proteins, *SEN* sensitivity, *SPE* specificity, *MALDI-TOF MS* matrix-assisted laser desorption/ionization time-of-flight mass spectrometry, *AbMB* antibody-conjugated magnetic beads

^a^represent markers extracted from extracellular vesicles

^b^BPD and HC

^c^benign pancreatic tumor, CP, and HC

^d^benign pancreatic tumor and pancreatitis

^e^HC, CP, and IPMN

^f^no history of cancer

^g^serous cyst adenoma and CP

^h^well-differentiated pancratic neuroendocrine tumor, pancreatic cystic lesions, choronic pancreatitis, and HC

^k^liver injury, pancreatitis, and cholangitis

^*^represent estimated value

*p* value indicates the *p* value of AUC

**Table 3:** Diagnostic performance of biomarker panels in extracellular vesicles for pancreatic cancer

Study	Country	study design	Cases vs Controls			Specimen	Stage	Controls Status	Detection Method	Panel	SEN%	SPE%	AUC	*P* Value
			**Number**	**Age**	**Male (%)**									
Madhavan, 2015 [43]	Germany	Case–control	75/45	/	/	Serum	I-IV	NC^b^	qPCR	panel A	**81**	**93**	**0.94**	/
									Flow cytometry	panel B	**96**	**100**	**0.99**	/
									qPCR/Flow cytometry	panel A/B	**100**	**93**	/	/
Yang, 2017 [47]^a^	USA	prospective	22/21	/	/	plasma	/	NC^b^	NPS chip	panel C	**86**	**86**	/	/
										panel D	**82**	**90**	/	/
										panel E	**86**	**81**	/	/
										panel F	**95**	**81**	/	/
Lewis, 2018 [50]	USA	Case–control	20/11	64/NA	/	Plasma	IIA- IIB	HC	ACE Immunoassay	panel G	94	91	0.99	/
			20/6	64/60	70/33	Plasma	II A- II B	BPD	ACE Immunoassay		81	78	0.81	/
Xiao, 2019 [79]	China	Case–control	24/26	59/45	42/85	Plasma	/	HC	Flow cytometry	panel H	76^e^	96^e^	0.90	/
			24/6	59/73	42/83	Plasma	/	CP	Flow cytometry		100^e^	66^e^	0.90	/
Yu, 2019 [85]^a^	China	Case–control	95/83	61/NA	57/66	Plasma	I-IV	NC^c^	ExLR-seq	panel I	**94**	**92**	**0.94**	/
			52/83	/	/		I-II				**88**	**92**	**0.91**	/
			35/83	/	/		I				**85**	**92**	**0.90**	/
			17/83	/	/		II				**94**	**92**	**0.93**	/
			43/83	/	/		III-IV				**100**	**92**	**0.97**	/
			95/40	61/53	57/70	Plasma	I-IV	CP	ExLR-seq		**94**	**90**	**0.95**	/
			52/40	/	/		I-II				**89**	**90**	**0.92**	/
			35/40	/	/		I				**86**	**90**	**0.91**	/
			17/40	/	/		II				**94**	**90**	**0.95**	/
			43/40	/	/		III-IV				**100**	**90**	**0.99**	/
			95/43	61/63	57/62	Plasma	I-IV	HC	ExLR-seq		**94**	**93**	**0.92**	/
Rodrigues, 2019 [33]^a^	USA	Case–control	20/12	/	45/25	serum		NC^f^	nanoparticle-and dyebased fluorescent immunoassay	panel Q	/	/	0.95	/
Reese, 2020 [31]	Germany	Case–control	56/33	/	64/52	Serum-EpCAM	II-IV	NC^c^	qPCR	panel J	64^e^	91^e^	0.84	0.0004
Zhou, 2020 [62]	China	Case–control	30/10	60/58	/	Plasma	I-IV	NC^d^	3D mircrofluidic chip	panel K	100^e^	100^e^	1.00	/
Wu, 2020 [61]	China	Case–control	30/10	62/51	60/80	serum	0-IV	CP	qPCR	panel L	93	80	/	/
Pu, 2020 [25]	China	Case–control	36/65	/	/	Plasma	I-IV	HC	cationic lipoplex nanoparticle	panel M	75 ^e^	80 ^e^	0.79	< 0.0001
Qin, 2021 [63]^a^	China	Case–control	44/27	/	50/44	Plasma	I-IV	HC	qPCR	panel N	75^e^	74^e^	**0.78**	/
										panel P	91^e^	74^e^	**0.89**	/
			44/40	/	50/65			CP		panel N	92^e^	40^e^	**0.71**	/
										panel P	64^e^	90^e^	**0.77**	/
			44/67		50/57			NC^c^		panel N	72^e^	63^e^	**0.70**	/
										panel P	53^e^	83^e^	**0.72**	/
Li, 2021 [76]^a^	China	Case–control	21/29	54/62	43/55	plasma	/	HC	AbMB-bioChol paltform	panel R	38^e^	93^e^	**0.74**	/
Wu, 2021 [71]	China	Case–control	284/117	/	59/62	plasma	I-IV	HC	ExLR-seq	panel S	80^e^	73^e^	0.86	/
			284/100	/	59/46			CP			64^e^	82^e^	**0.84**	/
			14/32	/	/		/	HC	RNA-seq	panel T	100	100	1.00	/
Verel-Y, 2021 [73]	Germany	Case–control	72/20	/	50/NA	Serum	I-IV	HC	bead-coupled FACS/qPCR	panel U	100	100	1.00	/
										panel V	91^e^	82^e^	0.93	/
Kim, 2021 [77]^a^	Korea	Case–control	20/20	61/51	70/50	plasma	I-III	CL	qPCR	panel W	38^e^	95^e^	0.69	/
										panel X	65^e^	99^e^	0.77	/
										panel Y	76^e^	75^e^	0.84	/
										panel Z	85^e^	81^e^	0.87	/
										panel 1	56^e^	95^e^	0.79	/
										panel 2	81	80	0.87	/
										panel 3	76	90	0.87	/
										panel 4	76	90	0.90	/
										panel 5	81	85	0.86	/
										panel 6	76	85	0.87	/
										panel 7	76	85	0.91	/
										panel 8	86	90	0.91	/
Kim, 2021 [77]^a^	Korea	Case–control	20/20	61/51	70/50	plasma	I-III	CL	qPCR	panel 9	76	90	0.93	/
										panel 10	81	85	0.91	/
										panel 11	67	90	0.81	/
										panel 12	76	90	0.90	/
										panel 13	86	90	0.94	/
										panel 14	90	90	0.95	/
										panel 15	86	90	0.94	/
										panel 16	81	85	0.92	/
										panel 17	86	90	0.91	/
										panel 18	76	90	0.94	/
										panel 19	90	85	0.94	/
										panel 20	81	90	0.96	/
										panel 21	86	90	0.94	/
										panel 22	90	90	0.96	/
										panel 23	90	85	0.95	/
										panel 24	90	90	0.96	/
										panel 25	86	90	0.95	/
										panel 26	86	85	0.96	/
										panel 27	90	90	0.97	/
Guo, 2021 [78]^a^	China	Case–control	27/15	57/43	63/60	plasma	IB-IV	CP	small RNA sequencing	panel 28	81	93	**0.88**	/
			30/18	63/44	63/72		IB-III			panel 29	/	/	0.94	/
										panel 30	/	/	0.94	/
										panel 31	/	/	0.94	/
										panel 32	/	/	0.94	/
										panel 33	/	/	0.94	/
										panel 34	/	/	0.94	/
Guo, 2021 [78]^a^	China	Case–control	30/18	63/44	63/72	plasma	IB-III	CP	small RNA sequencing	panel 35	/	/	0.93	/
										panel 36	/	/	0.93	/
										panel 37	/	/	0.93	/
										panel 38	/	/	0.93	/
										panel 39	/	/	0.93	/
										panel 40	/	/	0.93	/
										panel 41	/	/	0.92	/
										panel 42	/	/	0.92	/
										panel 43	/	/	0.92	/
										panel 44	/	/	0.92	/
										panel 45	/	/	0.92	/
										panel 46	/	/	0.92	/
										panel 47	/	/	0.92	/
										panel 48	/	/	0.92	/
										panel 49	/	/	0.91	/
										panel 50	/	/	0.91	/
										panel 51	/	/	0.91	/
										panel 52	/	/	0.91	/
										panel 53	/	/	0.91	/
										panel 54	/	/	0.91	/
										panel 55	/	/	0.91	/
										panel 56	/	/	0.91	/
										panel 57	/	/	0.91	/
										panel 58	/	/	0.91	/
										panel 59	/	/	0.90	/
										panel 60	/	/	0.90	/
Guo, 2021 [78]^a^	China	Case–control	30/18	63/44	63/72	plasma	IB-III	CP	small RNA sequencing	panel 61	/	/	0.90	/
										panel 62	/	/	0.90	/
										panel 63	/	/	0.90	/
										panel 64	/	/	0.90	/
										panel 65	/	/	0.90	/
										panel 66	/	/	0.90	/
										panel 67	/	/	0.90	/
										panel 68	/	/	0.90	/
										panel 69	/	/	090	/
										panel 70	/	/	0.90	/
										panel 71	/	/	0.90	/
										panel 72	/	/	0.90	/
										panel 73	/	/	0.90	/
										panel 74	/	/	0.90	/
										panel 75	/	/	0.90	/
										panel 76	/	/	0.89	/
										panel 77	/	/	0.89	/
										panel 78	/	/	0.89	/
										panel 79	/	/	0.89	/
										panel 80	/	/	0.89	/
										panel 81	/	/	0.89	/
										panel 82	/	/	0.89	/
										panel 83	/	/	0.89	/
										panel 84	/	/	0.89	/
										panel 85	/	/	0.89	/
										panel 86	/	/	0.89	/
Guo, 2021 [78]^a^	China	Case–control	30/18	63/44	63/72	plasma	IB-III	CP	small RNA sequencing	panel 87	/	/	0.89	/
										panel 88	/	/	0.89	/
										panel 89	/	/	0.89	/
										panel 90	/	/	0.88	/
										panel 91	/	/	0.88	/
										panel 92	/	/	0.88	/
										panel 93	/	/	0.88	/
										panel 94	/	/	0.88	/
										panel 95	/	/	0.88	/
										panel 96	/	/	0.88	/
										panel 97	/	/	0.88	/
										panel 98	/	/	0.88	/
										panel 99	/	/	0.88	/
										panel 100	/	/	0.88	/
										panel 101	/	/	0.88	/
										panel 102	/	/	0.88	/
										panel 103	/	/	0.87	/
										panel 104	/	/	0.87	/
										panel 105	/	/	0.87	/
										panel 106	/	/	0.87	/
										panel 107	/	/	0.87	/
										panel 108	/	/	0.87	/
										panel 109	/	/	0.87	/
										panel 110	/	/	0.86	/
										panel 111	/	/	0.86	/
										panel 112	/	/	0.86	/
Guo, 2021 [78]^a^	China	Case–control	30/18	63/44	63/72	plasma	IB-III	CP	small RNA sequencing	panel 113	/	/	0.86	/
										panel 114	/	/	0.86	/
										panel 115	/	/	0.85	/
										panel 116	/	/	0.85	/
										panel 117	/	/	0.85	/
										panel 118	/	/	0.84	/
										panel 119	/	/	0.83	/
										panel 120	/	/	0.83	/
										panel 121	/	/	0.83	/
										panel 122	/	/	0.83	/
										panel 123	/	/	0.83	/
										panel 124	/	/	0.82	/
										panel 125	/	/	0.82	/
										panel 126	/	/	0.822	/
										panel 127	/	/	0.82	/
										panel 128	/	/	0.82	/
										panel 129	/	/	0.81	/
										panel 130	/	/	0.81	/
										panel 131	/	/	0.80	/
										panel 132	/	/	0.79	/
										panel 133	/	/	0.77	/
										panel 134	/	/	0.76	/
										panel 135	/	/	0.76	/
										panel 136	/	/	0.76	/
										panel 137	/	/	0.75	/
										panel 138	/	/	0.74	/
Guo, 2021 [78]^a^	China	Case–control	30/18	63/44	63/72	plasma	IB-III	CP	small RNA sequencing	panel 139	/	/	0.74	/
										panel 140	/	/	0.74	/
										panel 141	/	/	0.72	/
										panel 142	/	/	0.72	/
										panel 143	/	/	0.72	/
										panel 144	/	/	0.71	/
										panel 145	/	/	0.71	/
										panel 146	/	/	0.71	/

SENs, SPEs and AUCs in bold fonts represent results from validation set (non-bold fonts represent results without validation)

*AUC* area under the curve, *ACE* alternating current electrokinetic, *AbMB* antibody-conjugated magnetic beads, *BPD* benign pancreatic disease, *CP* chronic pancreatitis, *CL* cholecytitis, *HC* healthy control, *FACS* Cartoon of protocol for flow cytometry, *NC* noncancerous, *NPS* nanoplasmonic sensor, *SEN* sensitivity, *SPE* specificity

^a^represent markers extracted from extracellular vesicles

^b^HC, CP, and benign pancreatic tumor

^c^HC and CP

^d^no history of cancer

^e^represent 估算值

^f^represent liver injury, pancreatitis, and cholangitis

Panel A: miR-1246, miR-4644, miR-3976, miR-4306; Panel B, CD44v6/Tspan8/EpCAM/CD104; panel C, EGFR/EPCAM/HER2/MUC1; Panel D, EGFR/EPCAM/GPC1/WNT2; Panel E, EGFR/EPCAM/MUC1/GPC1/WNT2; Panel F, EGFR/EPCAM/HER2/MUC1/GPC1/WNT2; Panel G, GPC1/CD63; Panel H, GPC1/CD82; Panel I, FGA/KRT19/HIST1H2BK/ITIH2/MARCH2/CLDN1/MAL2/TIMP1; panel Q, EpCAM/EphA2; Panel J, miR-200c/miR-200b; Panel K, miR-451a/21/10b/EphA2; Panel L, miR-21/210; Panel M, miR-21/10b; Panel N, FBXO7/MORF4L1//DDX17/TALDO1//AHNAK/TUBA1B; Panel P, FBXO7/MORF4L1//DDX17/TALDO1//AHNAK/TUBA1B//CD44/SETD3; panel R, EGFR/EpCAM/GPC1/EphA2; panel S, HIST2H2AA3/LUZP6/HLA-DRA; panel T, HIST2H2AA3/HIST1H4K/HLD-DRA/RN7SL1/LUZP6/FAM184B/FGF23/NEUROD2/miR663AHG/GPM6A; panel U, ADAM8/miR-720; panel V, ADAM8/miR-451; panel W, ITGA2/ITGAV/GPC1/miR-10b; panel X, ITGA2/ITGAV/GPC1/miR-21; panel Y, ITGA2/ITGAV/GPC1/miR-155; panel Z, ITGA2/ITGAV/GPC1/miR-429; panel 1, ITGA2/ITGAV/GPC1/miR-1290; panel 2, ITGA2/ITGAV/GPC1/miR-21/miR-155; panel 3, ITGA2/ITGAV/GPC1/miR-21/miR-429; panel 4, ITGA2/ITGAV/GPC1/miR-21/miR-1290; panel 5, ITGA2/ITGAV/GPC1/miR-21/miR-10b; panel 6, ITGA2/ITGAV/GPC1/miR-155/miR-429; panel 7, ITGA2/ITGAV/GPC1/miR-155/miR-1290; panel 8, ITGA2/ITGAV/GPC1/miR-155/miR10b; panel 9, ITGA2/ITGAV/GPC1/miR-429/miR-1290; panel 10, ITGA2/ITGAV/GPC1/miR-429/miR-10b; panel 11, ITGA2/ITGAV/GPC1/miR-1290/miR-10b; panel 12, ITGA2/ITGAV/GPC1/miR-21/miR-155/miR-429; panel 13, ITGA2/ITGAV/GPC1/miR-21/miR-155/miR-10b; panel 14, ITGA2/ITGAV/GPC1/miR-21/miR-155/miR-1290; panel 15, ITGA2/ITGAV/GPC1/miR-21/miR-429/miR-1290; panel 16, ITGA2/ITGAV/GPC1/miR-21/miR-429/miR-10b; panel 17, ITGA2/ITGAV/GPC1/miR-21/miR-1290/miR-10b; panel 18, ITGA2/ITGAV/GPC1/miR-155/miR-429/miR-1290; panel 19, ITGA2/ITGAV/GPC1/miR-155/miR-429/miR-10b; panel 20, ITGA2/ITGAV/GPC1/miR-155/miR-1290/miR-10b; panel 21, ITGA2/ITGAV/GPC1/miR-429/miR-1290/miR-10b; panel 22, ITGA2/ITGAV/GPC1/miR-21/miR-155/miR-429/miR-1290; panel 23, ITGA2/ITGAV/GPC1/miR-21/miR-155/miR-429/miR-10b; panel 24, ITGA2/ITGAV/GPC1/miR-21/miR-155/miR-1290/miR-10b; panel 25, ITGA2/ITGAV/GPC1/miR-21/miR-429/miR-1290/miR-10b; panel 26, ITGA2/ITGAV/GPC1/miR-155/miR-429/miR-1290/miR-10b; panel 27, ITGA2/ITGAV/GPC1/miR-21/miR-155/miR-429/miR-1290/miR-10b; panel 28, miR-95-3p/miR-26b-5p; panel 29, miR-95-3p/miR-3605-3p; panel 30, miR-95-3p/miR-128-3p; panel 31, miR-95-3p/miR-30d-5p; panel 32,miR-95-3p/miR-505-5p; panel 33,miR-95-3p/miR-148b-3p; panel 34, miR-95-3p/miR-342-5p; panel 35,miR-95-3p/miR-532-5p; panel 36, miR-95-3p/let-7 g-5p; panel 37, miR-95-3p/miR-151a-3p; panel 38, miR-95-3p/miR-181a-2-3p; panel 39,miR-95-3p/miR-550a-5p; panel 40, miR-95-3p/let-7b-5p; panel 41, miR-95-3p/miR-191-5p; panel 42,miR-95-3p/miR-92a-3p; panel 43, miR-95-3p/miR-941; panel 44, miR-95-3p/miR-106b-3p; panel 45, miR-95-3p/miR-7706; panel 46, miR-95-3p/miR-183-5p; panel 47,miR-95-3p/miR-25-5p; panel 48,miR-95-3p/miR-486-3p; panel 49,miR-95-3p/miR-3158-3p; panel 50, miR-95-3p/miR-7-5p; panel 51, miR-95-3p/miR-101-3p; panel 52,miR-95-3p/miR-210-3p; panel 53,miR-95-3p/miR-550a-3-5p; panel 54, miR-95-3p/miR-584-5p; panel 55,miR-95-3p/miR-140-3p; panel 56, miR-95-3p/miR-4732-5p; panel 57, miR-95-3p/miR-363-5p; panel 58,miR-95-3p/miR-4326; panel 59, miR-95-3p/miR-1294; panel 60,miR-95-3p/miR-486-5p; panel 61, miR-95-3p/miR-185-3p; panel 62,miR-95-3p/miR-4732-3p; panel 63, miR-95-3p/miR-92b-3p; panel 64,miR-95-3p/miR-423-5p; panel 65,miR-95-3p/miR-503-5p; panel 66, miR-95-3p/miR-1180-3p; panel 67, miR-95-3p/miR-25-3p; panel 68,miR-95-3p/miR-92b-5p; panel 69, miR-95-3p/miR-1284; panel 70,miR-95-3p/miR-17-5p; panel 71, miR-95-3p/miR-2110; panel 72, miR-95-3p/miR-24–2-5p; panel 73,miR-95-3p/miR-339-3p; panel 74,miR-95-3p/miR-660-5p; panel 75,miR-95-3p/miR-6842-3p; panel 76, miR-95-3p/let-7d-5p; panel 77, miR-95-3p/miR-30e-5p; panel 78, miR-95-3p/miR-628-3p; panel 79, miR-95-3p/miR-629-5p; panel 80, miR-95-3p/let-7i-5p; panel 81, miR-95-3p/miR-142-5p; panel 82,miR-95-3p/miR-182-5p; panel 83, miR-95-3p/miR-1908-5p; panel 84, miR-95-3p/miR-425-5p; panel 85,miR-95-3p/miR-942-5p; panel 86, miR-95-3p/miR-93-5p; panel 87, miR-95-3p/miR-363-3p; panel 88, miR-95-3p/miR-18a-3p; panel 89, miR-95-3p/miR-320a; panel 90, miR-95-3p/miR-421; panel 91, miR-95-3p/miR-501-3p; panel 92,miR-95-3p/let-7a-3p; panel 93, miR-95-3p/miR-16–2-3p; panel 94, miR-95-3p/miR-16-5p; panel 95,miR-95-3p/miR-130b-3p; panel 96, miR-95-3p/miR-3613-5p; panel 97, miR-95-3p/miR-451a; panel 98, miR-95-3p/miR-20b-5p; panel 99, miR-95-3p/miR-103a-3p; panel 100, miR-95-3p/miR-1224-5p; panel 101, miR-95-3p/miR-185-5p; panel 102, miR-95-3p/miR-20a-5p; panel 103, miR-95-3p/miR-186-5p; panel 104, miR-95-3p/miR-3615; panel 105, miR-95-3p/miR-7976; panel 106, miR-95-3p/miR-652-3p; panel 107, miR-95-3p/miR-107; panel 108, miR-95-3p/miR-181a-5p; panel 109, miR-95-3p/miR-15b-3p; panel 110, miR-95-3p/let-7b-3p; panel 111, miR-95-3p/miR-10b-5p; panel 112, miR-95-3p/miR-24-3p; panel 113, miR-95-3p/miR-106b-5p; panel 114, miR-95-3p/miR-15a-5p; panel 115, miR-95-3p/miR-197-3p; panel 116, miR-95-3p/miR-32-5p; panel 117, miR-95-3p/miR-450b-5p; panel 118, miR-95-3p/let-7e-5p; panel 119, miR-95-3p/miR-155-5p; panel 120, miR-95-3p/miR-361-5p; panel 121, miR-95-3p/miR-126-3p; panel 122, miR-95-3p/miR-484; panel 123, miR-95-3p/miR-30a-5p; panel 124, miR-95-3p/miR-27a-3p; panel 125, miR-95-3p/miR-29a-3p; panel 126, miR-95-3p/miR-335-5p; panel 127, miR-95-3p/miR-125a-5p; panel 128, miR-95-3p/miR-338-5p; panel 129, miR-95-3p/miR-139-5p; panel 130, miR-95-3p/miR-22-5p; panel 131, miR-95-3p/miR-23a-3p; panel 132, miR-95-3p/miR-382-5p; panel 133, miR-95-3p/miR-543; panel 134, miR-95-3p/miR-499a-5p; panel 135, miR-95-3p/miR-4433b-3p; panel 136, miR-95-3p/miR-1228-5p; panel 137, miR-95-3p/miR-99b-5p; panel 138, miR-95-3p/miR-143-3p; panel 139, miR-95-3p/miR-206; panel 140, miR-95-3p/miR-224-5p; panel 141, miR-95-3p/miR-10a-5p; panel 142, miR-95-3p/miR-223-3p; panel 143, miR-95-3p/miR-134-5p; panel 144, miR-95-3p/miR-485-5p; panel 145, miR-95-3p/miR-760; panel 146, miR-95-3p/miR-199b-3p

The isolation and detection methods of EVs were diversity, 15 studies used the commercial kits [24, 25, 46, 53, 60, 61, 64, 68, 70–72, 74, 83–85], 11 studies used ultracentrifugation [31, 43, 45, 58, 59, 63, 69, 73, 75, 79, 81], one study was not stated the isolation and detection method of EV [80], the remaining studies developed new isolation and detection techniques including ephrin type-A receptor 2/nanoplasmon-enhanced scattering (EphA2-EV-nPES assay) [34], PDA encapsulated antibody-reporter-Ag(shell)-Au(core) multilayer, surface-Enhanced Raman Scattering (chip-exosome-PEARL SERS immunosensor) [82], AC electrokinetic integrated biomarker assay [86], nanoplasmonic sensor assay (NPS chip) [47], lipid-polymer hybrid nanoparticle/catalyzed hairpin DNA circuit (LPHN-CHDC biochip) [44], immunogold transmission electron microscopy [30], Sequential size-exclusion chromatography (SSEC) [67], antibody-conjugated magnetic beads, bivalent cholesterol-modified RNA − DNA duplexes (AbMB-bioChol) platform [76], nanoparticle-and dyebased fluorescent immunoassay [33], immuno-capture using magnetic beads [77], and 3D microfluidic chip [62, 78] (Additional file 1).

### Quality assessment

**Fig. 2:**
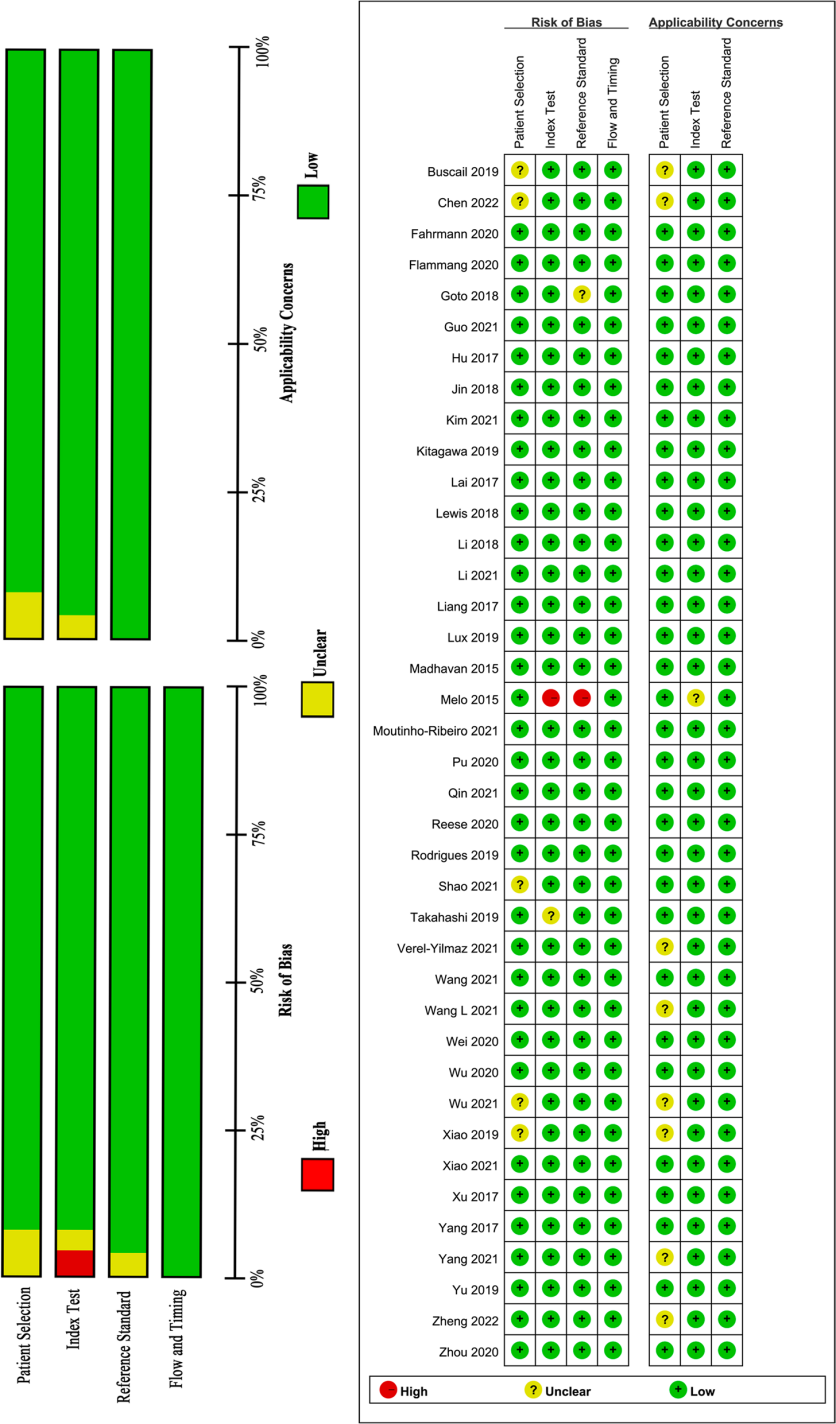
QUADAS-2 assessment. Risk of bias and applicability concerns summary (**A**) graph and (**B**) summary

We judged the risk of bias and applicability concerns of the included studies using QUADAS-2 evaluation tool, which were grouped four domains: participant selection, index test, reference standard, flow and timing. The outcomes of our assessment of methodological quality were summarized in Fig. 2. Twenty-seven studies were high quality studies with low risk of bias for all four domains. The risk of bias in the “index test” was high and the applicability concerns of the “index test” was unclear in Melo et al. 2015, as the index test might not be reproduced [30]; the index test of one study had unclear risk due to the unspecified of EVs isolation and detection method [80]; the risk of bias and applicability concerns of the “patient selection” were unclear in 6 studies [52, 56, 68, 71, 74], as the age and gender distribution was significant differences both in PC cases and control groups; The risk of bias and applicability concerns of the “reference standard” were unclear in Goto 2018, as the reference standard in this study was not clarified [84].

**Fig. 3:**
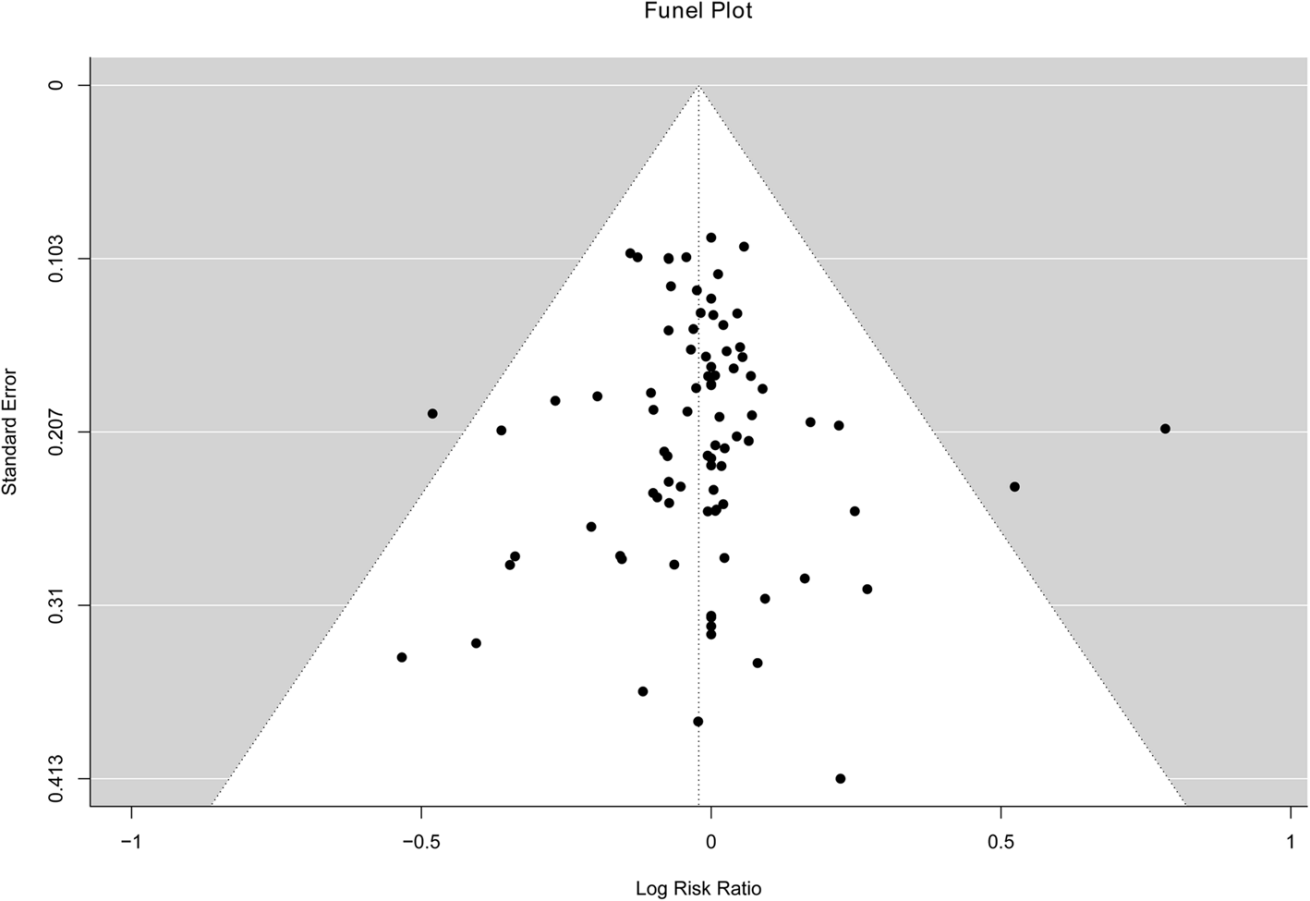
Funnel plot with 95% confidence limits

The results of egger’s test (Z = 0.28,* P* = 0.78) did not provided any evidence of publication bias. The funnel plot showed reasonably symmetrical, which also supports the results of egger’s test (Fig. 3).

### Diagnostic performance

In summary, a total number of 183 EV RNAs in 20 included studies were reported to be statistically significant in PC diagnosis and 161 EV RNAs were included in panels. Nine miRNAs (including miR-10b, miR-21, miR-451a, miR-106b, miR-155, miR-181a, miR-191, miR-1246, and miR-20a) were reported more than one times. Among them, miR-21 and miR-10b were the highest frequently reported RNA which was reported in 6 times. The expression direction of most EV miRNAs were up-regulate, apart from miR-122 [45], miR-let7 [45], miR-1226-3p [64], miR-19b-3p [72], miR-3158-3p [78], miR-4732-3p [78], miR-7706 [78], and miR-486-3p [78] were down-regulate (Additional file 2). The expression directions of miR-192-5p [59] and miR-196b [46] were not reported (Additional file 2). For individual EV RNAs, the median reported sensitivity and specificity were 82% (from 42 to 100%) and 90% (from 63 to 100%), respectively. Both sensitivity and specificity of 52% EV RNAs exceeded 80%. One study conducted validation test by Hu et al., which validated EV GPC1-mRNA as a good diagnostic biomarker for PC, with the sensitivity, specificity, and AUC were 95%, 93% and 0.94, respectively [44].

A total of 177 EV proteins reported in 13 studies were statistically significant for PC diagnosis, 19 EV proteins were included in panels. GPC1 was the most frequently reported EV protein with median sensitivity and specificity of 72% (from 43 to 100%) and 86% (from 52 to 100%), respectively, which was reported in nine times. And the following were EphA2, EGFR, and EPCAM (Additional file 3). For individual EV proteins, the median reported sensitivity and specificity were 64% (36–100%) and 85% (52–100%), respectively. Both sensitivity and specificity of 20% EV proteins exceeded 80%.

172 EV biomarker panels in 16 studies were reported and 12 panels were verified by independent validation among six studies (Table 3). Most panels showed powerful diagnostic accuracy with median sensitivity and specificity of 82% (from 38 to 100%) and 90% (from 63 to 100%), respectively. Both the sensitivity and specificity of 63% panels exceeded 80%.

Nine studies reported diagnosis performance of EV biomarkers (17 individual biomarkers and 2 panels) for early stage (stage I-II) PC, two of individual biomarkers were conducted independent validation tests. And across these two validation studies, both the sensitivity and specificity of EV biomarkers for early stage (stage I-II) PC was more than 85% [34]. The median reported sensitivity and specificity for early stage PC were 94% (from 67 to 100%) and 86% (from 73 to 100%), respectively. Both sensitivity and specificity of 68% EV biomarkers exceeded 80%.

### Results of meta-analysis

**Fig. 4:**
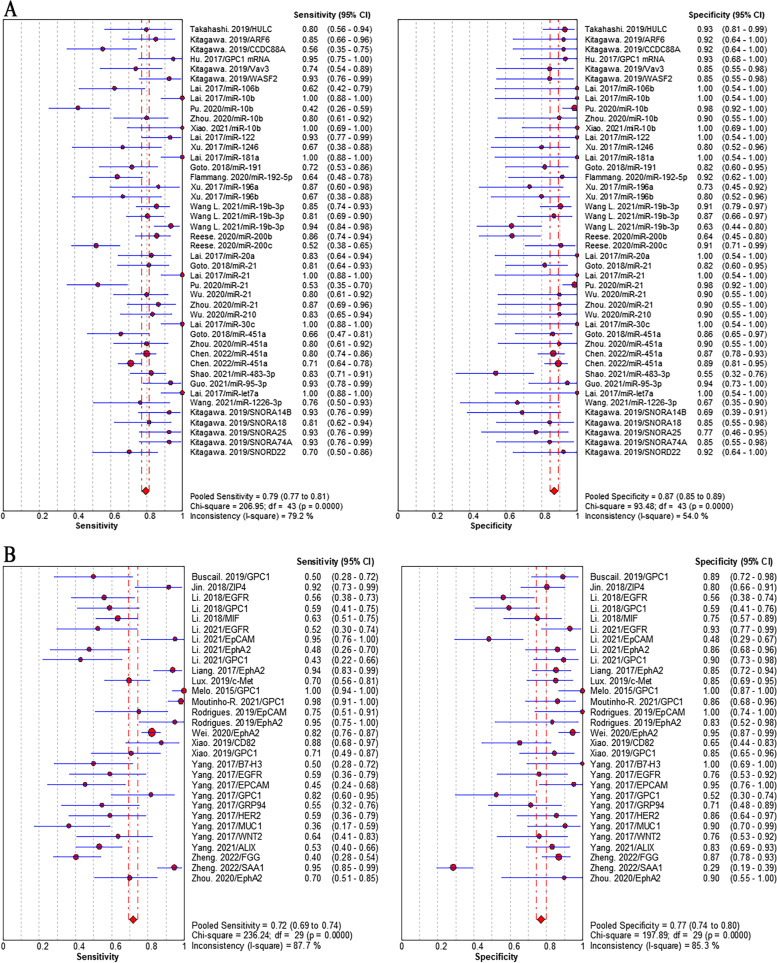
Pooled sensitivity and specificity of EV biomarkers for pancreatic cancer diagnosis. **A** individual EV RNAs, (**B**) individual EV proteins

**Fig. 5:**
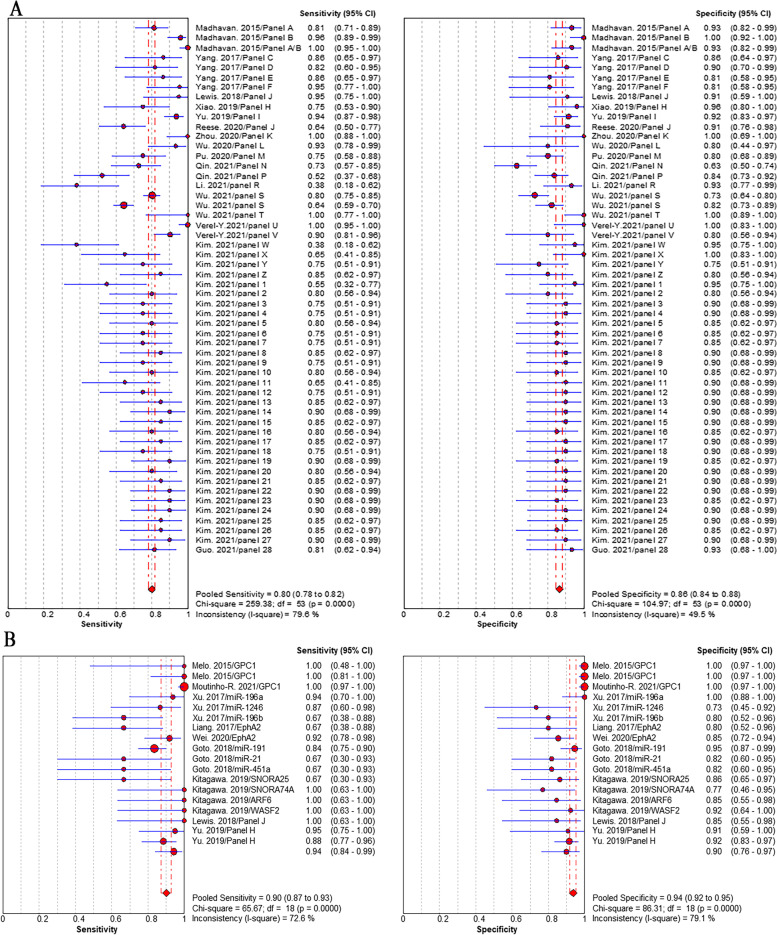
Pooled sensitivity and specificity of individual EV biomarkers for pancreatic cancer diagnosis. **A** EV biomarker panels, (**B**) EV biomarkers for early stage PC

Studies that reported individual EV biomarkers for PC diagnosis were performed meta-analyses. Overall, 32 individual EV RNAs investigated in 16 studies and 16 individual EV proteins investigated in 13 studies were included in the meta-analyses. The sensitivity (± 95% confidence intervals) and specificity (± 95% confidence intervals) for each individual EV RNAs and each individual EV proteins were depicted in the corresponding forest plots. The pooled sensitivity and specificity of individual EV RNAs was 79% (95% CI: 77–81%) and 87% (95% CI: 85–89%), respectively (Fig. 4A). The pooled sensitivity and specificity of individual EV proteins was 72% (95% CI: 69–74%) and 77% (95% CI: 74–80%), respectively (Fig. 4B). The pooled sensitivity and specificity of EV biomarker panels were 80% (95% CI: 78–82%) and 86% (95% CI: 84–88%), respectively (Fig. 5A). We separately analyzed the diagnostic performance of EV RNA panels, EV protein panels, and EV RNA combined with protein panels, the results showed that EV RNA combined with protein panels had higher diagnosis value than those of EV RNA panels or EV protein panels. The pooled sensitivity and specificity were 84% (95% CI: 78–82%) and 89% (95% CI: 84–88%) for RNA combined with protein panels, 76% (95% CI: 73–78%) and 82% (95% CI: 79–85%) for RNA panels, and 85% (95% CI: 80–89%) and 91% (95% CI: 86–95%) for protein panels, respectively (addition file 4). Positively, we focused on the diagnosis value of EV biomarkers for early stage (stage I and II) PC. The results showed that the pooled sensitivity and specificity were 90% (95% CI: 87–93%) and 94% (95% CI: 92–95%), respectively (Fig. 5B).

**Fig. 6:**
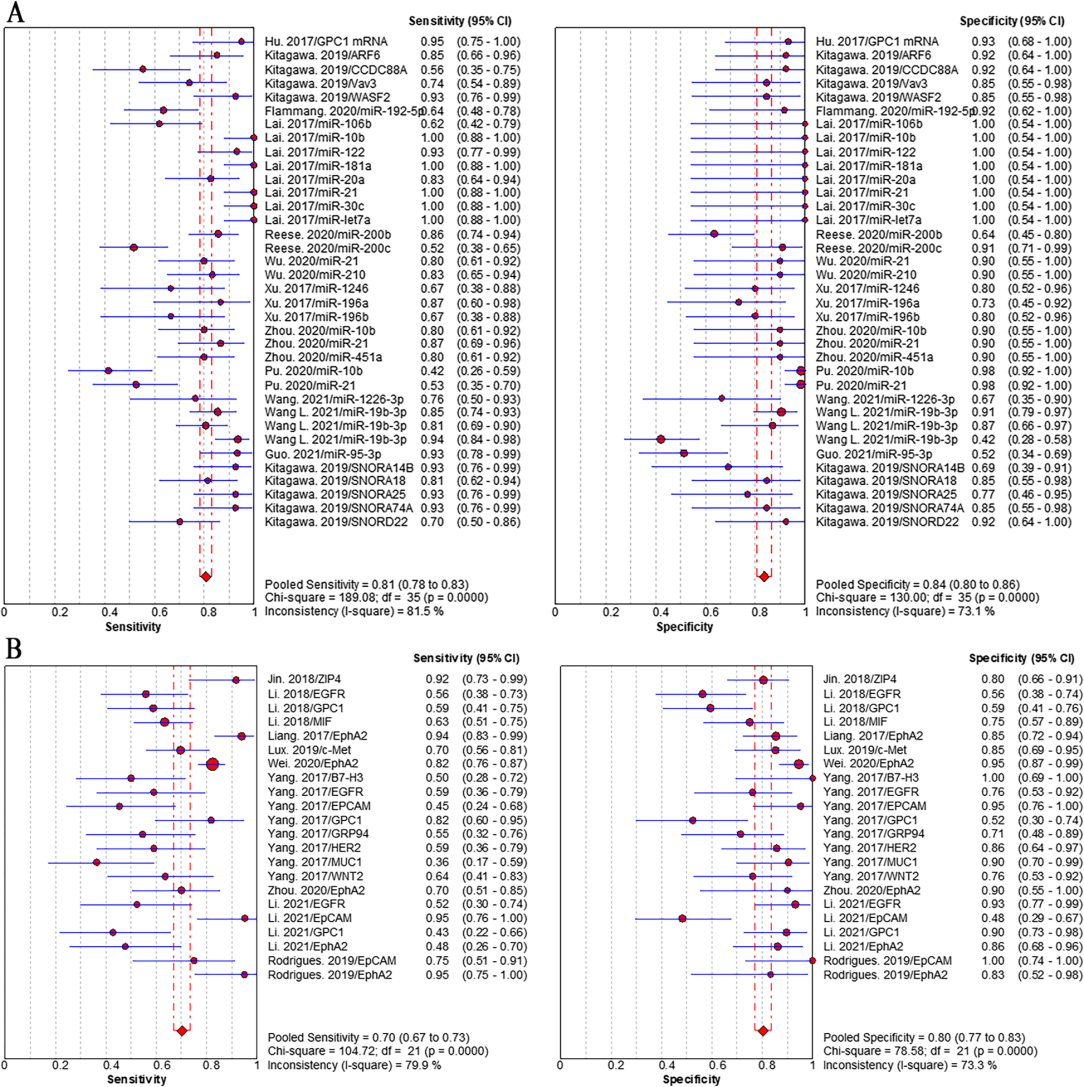
Pooled sensitivity and specificity of EV biomarkers for pancreatic cancer diagnosis in sensitivity analysis according to high quality studies. **A** individual EV RNAs, (**B**) individual EV proteins

**Fig. 7:**
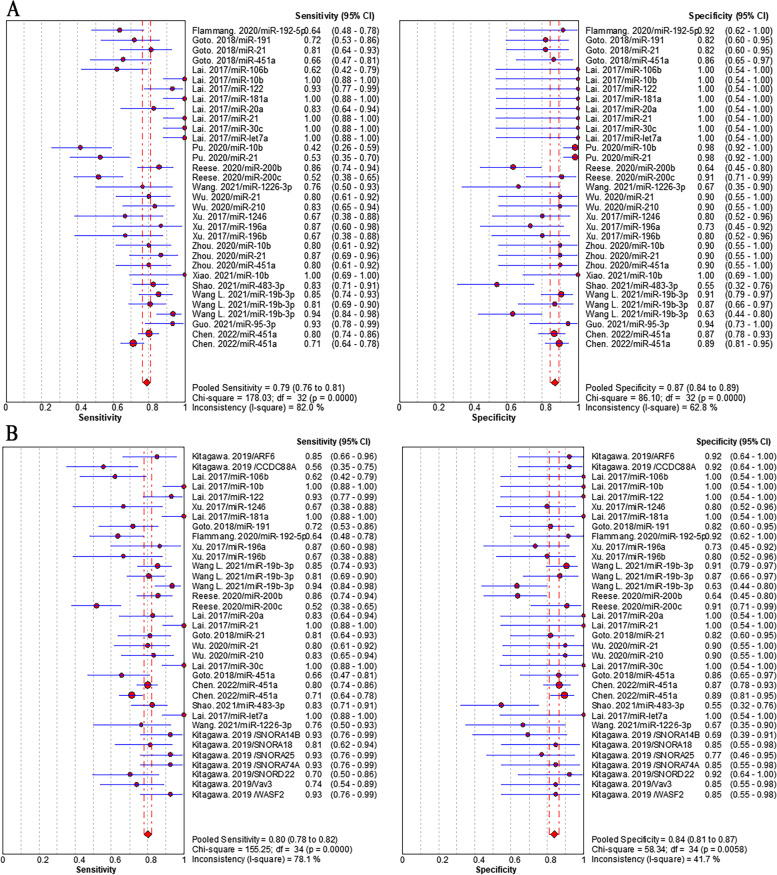
Pooled sensitivity and specificity of EV biomarkers for pancreatic cancer diagnosis in subgroup analyses. **A** individual EV miRNAs, (2) individual EV RNAs detected by qPCR

Sensitivity analysis in according to the high quality studies failed to demonstrate a change in the pooled sensitivity and specificity (RNAs: 81% (95%CI: 78–83%) and 84% (95%CI: 80–86%), Fig. 6A; Proteins: 70% (95%CI: 67–73%) and 80% (95% CI: 77–83%), Fig. 6B) for PC diagnosis. In subgroup analysis, we did not observe notable differences in pooled sensitivity and specificity of miRNAs (79% (95% CI: 76–81%) and 87% (95% CI: 84–89%), Fig. 7A) *vs* those of the whole RNAs (79% (95% CI: 77–81%) and 87% (95% CI: 85–89%)). We also found the similar pooled sensitivity and specificity between RNAs detected by qPCR (80% (95% CI: 78–82%) and 84% (95% CI: 81–87%), Fig. 7B) and those of the whole RNAs. It indicated that the heterogeneity in sensitivity and subgroup analyses was similar to the whole analysis.

## Discussion

A systematic understanding of diagnosis performance of tumor-related moleculars in circulation EVs is critical for PC screening and earliest detection. The present systematic review and meta-analysis has assembled the diagnostic performance of 183 EV RNAs, 177 EV proteins, and 172 EV biomarker panels based on serum/plasma for diagnosing PC in 39 studies from 2015 to 2022. The control groups selected noncancerous population containing healthy population, benign pancreatic disease, IPMN, serious cystadenoma, and nonpancreatic benign diseases. MiR-21 and miR-10b were the highest frequently reported EV RNA for PC diagnosis. GPC1 and EphA2 were mostly reported EV proteins for PC diagnosis. Both the EV RNAs and the EV proteins in high quality studies showed moderate diagnostic values and the EV panels revealed good diagnostic values for PC. Especially, the EV RNA combined protein panels had great diagnostic performances for PC with the pooled sensitivity of 84% and specificity of 89%. Surprisingly, for early stage PC the EV biomarkers showed excellent diagnostic performance, the sensitivity and specificity were 90% and 94%. Respectively. Overall, available studies highlighted the diagnostic performance of EV biomarkers to differentiate PC from noncancerous and further researches will be needed to validate the diagnostic value of EV biomarkers as noninvasive, effective, specific screening tools for PC diagnosis in general population.

EV GPC1 and EV EphA2 were the mostly frequent reported diagnostic proteins for PC in present study. GPC1 was discovered in two prospective studies [47, 75], one of which was conducted a blind validation test [47]. In 2015 Nature, EV GPC1 was demonstrated extremely surprising diagnostic accuracy for diagnosing PC, the AUC value reached 1.00 for all stage PC, and the validation test got consistent results that both the sensitivity and specificity of GPC1 for PC diagnosis was 100% [30]. This conclusion attracted highly attention worldwide at a time, but other researchers found inconsistent results at later [30]. A blind prospective study by Yang et al. reported that EV GPC1 had a good sensitivity for PC diagnosis, but the specificity poorly was 52% [47]. In 2018, Li et al. used exosome-based GPC1 to diagnose PC by an ultrasensitive immunoassay method. Although exosome GPC1 could distinguish PC patients from healthy controls, the diagnosis ability was limited and the sensitivity and specificity was 59% and 58%, respectively [82]. Reccently, Xiao et al. established a simple reproducible analysis method for detection exosome GPC1 for PC screening in chinese cohort, the results showed a excellent diagnostic performance with 100% sepcificity and 100% sensitivity [79]. We speculated that the inconsistent diagnosis performance of EV GPC1 for PC might be related to the non-standardized isolation and detection methods, nonuniform experimental procedure, different sample collection protocol, and various data analysis methods. Although the diagnostic performance of EV GPC1 for PC is varied, EV GPC1 indeed is a promising noninvasive biomarker for early diagnosis PC. Almost all of studies reported the diagnosis performance of EV EphA2 revealed great diagnosis value with AUC value equal or beyond 0.85, except one study with AUC value of 0.64 [76]. The result of a large cohort validated study demonstrated that for both I-II stage PC and all stage PC EV EphA2 showed strongly diagnostic efficiency. Especially, EV EphA2 could accurately distinguish PC patients with I-II stage from healthy controls (AUC = 0.96) or pancreatitis (AUC = 0.93) [34].

EV miRNAs recently have raised more interest as novel noninvasive biomarkers for malignant tumors. In our subgroup analysis EV miRNAs appeared powerful diagnosis capacity for PC with both sensitivity and specificity exceeded 80%, which was consistent with that of the whole EV RNAs. EV miR-21 was the most frequency reported miRNA and was significantly high expressed in PC with consistent direction in all five studies. EV miR-21 performed outstanding specificity for PC diagnosis, excepting in study by Goto et al. the specificity was 81%, the specificity value was all greater than 90% in the remaining studies and even two studies showed a specificity of 100% [25, 45, 61, 62, 84]. Aberrant expressed miR-21 related to the development and metastatic of malignant tumors and impacted signal transducer and activator of transcription 3 (STAT3), epidermal growth factor receptor (EGFR), transforming growth factor-β, and the p53 pathway in malignant tumor progression [87–90]. MiR-10b high expressed in PC and played important role in various malignant cancers, including PC. For example, PC patients had significantly higher expression level of miR-10b than healthy controls or pancreatitis and increased miR-10b expression in PC patients indicated tumor aggressiveness [91–94]; Abnormal expressed miR-10b promoted hepatocellular carcinoma cell proliferation and invasion and high expression level of EV miR-10b was related to advanced tumor [95]. However, there are number of challenges in using EV miRNAs as diagnostic biomarkers for PC, such as racial differences [96], unstandardized sample preparation procession [97], and nonuniform miRNA extraction kits [98]. Continued refinement of EV miRNAs techniques may help identify specific EV miRNAs for screening PC, which offers as promising non-invasive biomarkers for PC diagnosis in clinical practical.

In present study, we found EV biomarker panels showed high diagnosis value for PC, the pooled sensitivity and specificity was 80% and 86%, respectively, which showed a small advantage diagnosis performance than the whole individual EV RNAs and individual EV proteins. In previous studies, compared with the corresponding individual markers the diagnostic accuracy of either protein panels or miRNA panels was not to show any advantage [25, 61, 62, 79, 82]. However, combining EV RNAs with EV proteins, the diagnosis performance for PC was significant greater than that of the corresponding individual biomarkers. For example, both the sensitivity and specificity of EV panel composed by EphA2, miR-451a, miR-21, and miR-10b reached 100% [62]; Another panel consisted by EV miRNAs and EV proteins exhibited great diagnosis efficiency for PC with sensitivity of 100% and specificity of 93% [43]. In present study, we also found the diagnostic value of the EV RNA combined with protein panels showed good diagnostic performance, which was similar to the EV protein panels and a slight greater advantage than the EV RNA panels. Moreover, combined EV RNAs and EV proteins with conventional CA199 also significantly improved the diagnostic accuracy for PC. EV miR-200b and 200c and EpCAM combined with CA199 entailed a diagnostic accuracy of 97%, yielding sensitivity of 92% and specificity of 100% [31]; Therefore, the combine of novel EV biomarkers with conventional CA199 might be explored to increase diagnostic accuracy for PC and can be as noninvasive and low-cost diagnosis methods for PC screening in the future.

Due to high stability and providing specific information from original tumor of RNAs and proteins in EVs, EV RNAs and proteins have attracted considerable attention as noninvasive biomarkers for cancer diagnosis. By far, the analysis of EVs was separated into isolation and detection two steps. Multistep ultracentrifugation is the recommended standard method for EVs isolation, which increased the complexity of the operational process and was time-consuming and expensive, and the centrifugation time and force was nonuniform inducing major difference of EV purity and isolation rate [99]. Most included studies in this systematic review underwent the centrifugation step, but the centrifugation time and force are variable, parts of studies [85] even used once time centrifugation, which greatly affected the isolation concentration of EVs and further impacted the expression level of molecules contained in EVs. For decades, significant progression of EVs isolation and detection techniques has been made and two kinds of microfluidic-based techniques, immunoaffinity-based and size-based platforms, have been reported as most practical solutions to isolate and detect EV biomarkers [34, 44, 47, 62]. Microfluidics-based techniques provide an effective method to integrate the EV isolation and detection into a single chip, which overcome the abovementioned disadvantage [100]. In current study, six studies used microfluidic-based techniques to analysis the expression levels of EV biomarkers, such as 3D microfluidic chip, LPHN-CHDC biochip, ACE integrated biomarker assay, chip-exosome-PEARL SERS immunosensor, surface-Enhanced Raman Scattering, NPS chip, EphA2-EV-nPES assay, SSEC, AbMB-bioChol platform, nanoparticle-and dyebased fluorescent immunoassay, and immuno-capture using magnetic beads. Although microfluidic-based techniques show great advances in EVs isolation and detection, it need sufficient clinical samples to validate the application, accuracy, stability, and reproduction.

The heterogeneity of the present study ranged from mild to severe, neither subgroup analysis nor sensitivity analyses failed to reduce the degree of heterogeneity. Furthermore, we analyzed the pooled diagnostic value of EV biomarkers for early stage PC, the results also did not have impact on heterogeneity. We hypothesized that the sources of heterogeneity might be related to the various methods of EVs isolation and detection, sample selection protocol, and demographic or geographic different of study populations. In addition, the number of samples of case and control groups widely ranged from 6 to 284, which also contributed to the heterogeneity. Although the heterogeneity might limit the reliability of the pooled results in present study, the results of our systematic review and meta-analyses have a certain guide to pick up noninvasive and effective early diagnosis biomarkers for PC.

## Conclusions

In this systematic review and meta-analysis, we found circulation based EV biomarkers exhibited considerable diagnosis performance for PC. EV RNAs combined with EV proteins showed appealing higher diagnosis efficiency. Especially, for early stage PC the EV biomarkers revealed excellent diagnostic performance. However, the deficiency of technologies that can effectively isolation and detection EV biomarkers limit the application of EV biomarkers in clinical practice to some extent, highlighting the need for high-quality reproduction researches in this area as well a need for promising accuracy EV biomarkers for PC diagnosis and screening in larger sample prospective cohort.

## Supplementary Information

**Additional file 1. **Protocols of blood exosomes detection.

**Additional file 2. **Summary of studies reporting significant associations of RNAs in pancreatic cancer.

**Additional file 3. **Summary of studies reporting significant associations of proteins in pancreatic cancer.

**Additional file 4. **Pooled sensitivity and specificity of EV biomarker panels for pancreatic cancer diagnosis.

## Data Availability

All data generated or analyzed during this study are included in this published article and its supplementary information files.

## Abbreviations

EV: Extracellular vesicle
PC: Pancreatic cancer
IPMN: Intraductal papillary mucinous neoplasm
CP: Chronic pancreatitis
AUC: Area under the curve
ROC: Receiver operator characteristic
CA199: Carbohydrate antigen 199
QUADAS-2: Quality Assessment of Diagnostic Accuracy Studies 2
MP: Membrane protein; nMP non-membrane proteins
CI: Confidence Interval
EphA2-EV-nPES assay: Ephrin type-A receptor 2/nanoplasmon-enhanced scattering
chip-exosome-PEARL SERS immunosensor: PDA encapsulated antibody-reporter-Ag(shell)-Au(core) multilayer, surface-Enhanced Raman Scattering
LPHN-CHDC biochip: Lipid-polymer hybrid nanoparticle/catalyzed hairpin DNA circuit
NPS chip: Nanoplasmonic sensor assay
SSEC: Sequential size-exclusion chromatography
AbMB-bioChol: Antibody-conjugated magnetic beads, bivalent cholesterol-modified RNA − DNA duplexes
