# Supplementary material for: Extracellular vesicle biomarkers for pancreatic cancer diagnosis: a systematic review and meta-analysis
Source: BMC Cancer. 2022 May 23;22:573. doi: 10.1186/s12885-022-09463-x (PMC9125932; doi:10.1186/s12885-022-09463-x)
Supplement: Supplementary file 2 — Additional file 2. Summary of studies reporting significant associations of RNAs in pancreatic cancer. [file 12885_2022_9463_MOESM2_ESM.docx]

| **Additional file 2. Summary of studies reporting significant associations of RNAs in pancreatic cancer** | | | | | | | | | | | | | | | | | | | | | | | | |
| --- | --- | --- | --- | --- | --- | --- | --- | --- | --- | --- | --- | --- | --- | --- | --- | --- | --- | --- | --- | --- | --- | --- | --- | --- |
|  | **Ref.** | | | | | | | | | | | | | | | | | | | | | | |  |
| **RNA** | **Chen, 2022** | **Flammang, 2020** | **Goto, 2018** | **Guo, 2021** | **Hu, 2017** | **Kim, 2021** | **Kitagawa, 2019** | **Lai, 2017** | **Madhavan, 2015** | **Pu, 2020** | **Qin, 2021** | **Reese, 2020** | **Shao, 2021** | **Takahashi, 2019** | **Verel-Y,2021** | **Wang L, 2021** | **Wang, 2021** | **Wu, 2020** | **Wu, 2021** | **Xiao, 2021** | **Xu, 2017** | **Yu, 2019** | **Zhou, 2020** | **number of studies** |
| miR-10b |  |  |  | △ |  | △ |  | ↑○ |  | ↑△ |  |  |  |  |  |  |  |  |  | ↑○ |  |  | ↑△ | 6 |
| miR-21 |  |  | ↑○ |  |  | △ |  | ↑○ |  | ↑△ |  |  |  |  |  |  |  | ↑△ |  |  |  |  | ↑△ | 6 |
| miR-451a | ↑○ |  | ↑○ | ↑△ |  |  |  |  |  |  |  |  |  |  |  |  |  |  |  |  |  |  | ↑△ | 4 |
| miR-106b |  |  |  | △ |  |  |  | ↑○ |  |  |  |  |  |  |  |  |  |  |  |  |  |  |  | 2 |
| miR-1246 |  |  |  |  |  |  |  |  | △ |  |  |  |  |  |  |  |  |  |  |  | ↑○ |  |  | 2 |
| miR-155 |  |  |  | △ |  | △ |  |  |  |  |  |  |  |  |  |  |  |  |  |  |  |  |  | 2 |
| miR-181a |  |  |  | △ |  |  |  | ↑○ |  |  |  |  |  |  |  |  |  |  |  |  |  |  |  | 2 |
| miR-191 |  |  | ↑○ | △ |  |  |  |  |  |  |  |  |  |  |  |  |  |  |  |  |  |  |  | 2 |
| miR-20a |  |  |  | △ |  |  |  | ↑○ |  |  |  |  |  |  |  |  |  |  |  |  |  |  |  | 2 |
| miR-10a-5p |  |  |  | ↑△ |  |  |  |  |  |  |  |  |  |  |  |  |  |  |  |  |  |  |  | 1 |
| AHNAK |  |  |  |  |  |  |  |  |  |  | △ |  |  |  |  |  |  |  |  |  |  |  |  | 1 |
| ARF6 |  |  |  |  |  |  | ↑○ |  |  |  |  |  |  |  |  |  |  |  |  |  |  |  |  | 1 |
| CCDC88A |  |  |  |  |  |  | ↑○ |  |  |  |  |  |  |  |  |  |  |  |  |  |  |  |  | 1 |
| CD44 |  |  |  |  |  |  |  |  |  |  | △ |  |  |  |  |  |  |  |  |  |  |  |  | 1 |
| CLDN1 |  |  |  |  |  |  |  |  |  |  |  |  |  |  |  |  |  |  |  |  |  | △ |  | 1 |
| DDX17 |  |  |  |  |  |  |  |  |  |  | △ |  |  |  |  |  |  |  |  |  |  |  |  | 1 |
| FAM184B |  |  |  |  |  |  |  |  |  |  |  |  |  |  |  |  |  |  | △ |  |  |  |  | 1 |
| FBXO7 |  |  |  |  |  |  |  |  |  |  | △ |  |  |  |  |  |  |  |  |  |  |  |  | 1 |
| FGA |  |  |  |  |  |  |  |  |  |  |  |  |  |  |  |  |  |  |  |  |  | △ |  | 1 |
| FGF23 |  |  |  |  |  |  |  |  |  |  |  |  |  |  |  |  |  |  | △ |  |  |  |  | 1 |
| GPC1 mRNA |  |  |  |  | ↑○ |  |  |  |  |  |  |  |  |  |  |  |  |  |  |  |  |  |  | 1 |
| GPM6A |  |  |  |  |  |  |  |  |  |  |  |  |  |  |  |  |  |  | △ |  |  |  |  | 1 |
| HIST1H2BK |  |  |  |  |  |  |  |  |  |  |  |  |  |  |  |  |  |  |  |  |  | △ |  | 1 |
| HIST1H4K |  |  |  |  |  |  |  |  |  |  |  |  |  |  |  |  |  |  | △ |  |  |  |  | 1 |
|  |  |  |  |  |  |  |  |  |  |  |  |  |  |  |  |  |  |  |  |  |  |  |  |  |
| **Additional file 2 continue.** | | | | | | | | | | | | | | | | | | | | | | | | |
|  | **Ref.** | | | | | | | | | | | | | | | | | | | | | | |  |
| **RNA** | **Chen, 2022** | **Flammang, 2020** | **Goto, 2018** | **Guo, 2021** | **Hu, 2017** | **Kim, 2021** | **Kitagawa, 2019** | **Lai, 2017** | **Madhavan, 2015** | **Pu, 2020** | **Qin, 2021** | **Reese, 2020** | **Shao, 2021** | **Takahashi, 2019** | **Verel-Y,2021** | **Wang L, 2021** | **Wang, 2021** | **Wu, 2020** | **Wu, 2021** | **Xiao, 2021** | **Xu, 2017** | **Yu, 2019** | **Zhou, 2020** | **number of studies** |
| HIST2H2AA3 |  |  |  |  |  |  |  |  |  |  |  |  |  |  |  |  |  |  | △ |  |  |  |  | 1 |
| HLA-DRA |  |  |  |  |  |  |  |  |  |  |  |  |  |  |  |  |  |  | △ |  |  |  |  | 1 |
| HULC |  |  |  |  |  |  |  |  |  |  |  |  |  | ↑○ |  |  |  |  |  |  |  |  |  | 1 |
| ITIH2 |  |  |  |  |  |  |  |  |  |  |  |  |  |  |  |  |  |  |  |  |  | △ |  | 1 |
| KRT19 |  |  |  |  |  |  |  |  |  |  |  |  |  |  |  |  |  |  |  |  |  | △ |  | 1 |
| let-7a-3p |  |  |  | △ |  |  |  |  |  |  |  |  |  |  |  |  |  |  |  |  |  |  |  | 1 |
| let-7b-3p |  |  |  | △ |  |  |  |  |  |  |  |  |  |  |  |  |  |  |  |  |  |  |  | 1 |
| let-7b-5p |  |  |  | △ |  |  |  |  |  |  |  |  |  |  |  |  |  |  |  |  |  |  |  | 1 |
| let-7d-5p |  |  |  | △ |  |  |  |  |  |  |  |  |  |  |  |  |  |  |  |  |  |  |  | 1 |
| let-7e-5p |  |  |  | △ |  |  |  |  |  |  |  |  |  |  |  |  |  |  |  |  |  |  |  | 1 |
| let-7g-5p |  |  |  | △ |  |  |  |  |  |  |  |  |  |  |  |  |  |  |  |  |  |  |  | 1 |
| let-7i-5p |  |  |  | △ |  |  |  |  |  |  |  |  |  |  |  |  |  |  |  |  |  |  |  | 1 |
| LUZP6 |  |  |  |  |  |  |  |  |  |  |  |  |  |  |  |  |  |  | △ |  |  |  |  | 1 |
| MAL2 |  |  |  |  |  |  |  |  |  |  |  |  |  |  |  |  |  |  |  |  |  | △ |  | 1 |
| miR-101-3p |  |  |  | △ |  |  |  |  |  |  |  |  |  |  |  |  |  |  |  |  |  |  |  | 1 |
| miR-103a-3p |  |  |  | △ |  |  |  |  |  |  |  |  |  |  |  |  |  |  |  |  |  |  |  | 1 |
| miR-106b-3p |  |  |  | △ |  |  |  |  |  |  |  |  |  |  |  |  |  |  |  |  |  |  |  | 1 |
| miR-107 |  |  |  | △ |  |  |  |  |  |  |  |  |  |  |  |  |  |  |  |  |  |  |  | 1 |
| miR-1180-3p |  |  |  | △ |  |  |  |  |  |  |  |  |  |  |  |  |  |  |  |  |  |  |  | 1 |
| miR-122 |  |  |  |  |  |  |  | ↓○ |  |  |  |  |  |  |  |  |  |  |  |  |  |  |  | 1 |
| miR-1224-5p |  |  |  | △ |  |  |  |  |  |  |  |  |  |  |  |  |  |  |  |  |  |  |  | 1 |
| miR-1226-3p |  |  |  |  |  |  |  |  |  |  |  |  |  |  |  |  | ↓○ |  |  |  |  |  |  | 1 |
| miR-1228-5p |  |  |  | △ |  |  |  |  |  |  |  |  |  |  |  |  |  |  |  |  |  |  |  | 1 |
| miR-125a-5p |  |  |  | △ |  |  |  |  |  |  |  |  |  |  |  |  |  |  |  |  |  |  |  | 1 |
| miR-126-3p |  |  |  | △ |  |  |  |  |  |  |  |  |  |  |  |  |  |  |  |  |  |  |  | 1 |
|  |  |  |  |  |  |  |  |  |  |  |  |  |  |  |  |  |  |  |  |  |  |  |  |  |
|  |  |  |  |  |  |  |  |  |  |  |  |  |  |  |  |  |  |  |  |  |  |  |  |  |
| **Additional file 2 continue** | | | | | | | | | | | | | | | | | | | | | | | | |
|  | **Ref.** | | | | | | | | | | | | | | | | | | | | | | |  |
| **RNA** | **Chen, 2022** | **Flammang, 2020** | **Goto, 2018** | **Guo, 2021** | **Hu, 2017** | **Kim, 2021** | **Kitagawa, 2019** | **Lai, 2017** | **Madhavan, 2015** | **Pu, 2020** | **Qin, 2021** | **Reese, 2020** | **Shao, 2021** | **Takahashi, 2019** | **Verel-Y,2021** | **Wang L, 2021** | **Wang, 2021** | **Wu, 2020** | **Wu, 2021** | **Xiao, 2021** | **Xu, 2017** | **Yu, 2019** | **Zhou, 2020** | **number of studies** |
| miR-128-3p |  |  |  | △ |  |  |  |  |  |  |  |  |  |  |  |  |  |  |  |  |  |  |  | 1 |
| miR-1284 |  |  |  | △ |  |  |  |  |  |  |  |  |  |  |  |  |  |  |  |  |  |  |  | 1 |
| miR-1290 |  |  |  |  |  | △ |  |  |  |  |  |  |  |  |  |  |  |  |  |  |  |  |  | 1 |
| miR-1294 |  |  |  | △ |  |  |  |  |  |  |  |  |  |  |  |  |  |  |  |  |  |  |  | 1 |
| miR-130b-3p |  |  |  | △ |  |  |  |  |  |  |  |  |  |  |  |  |  |  |  |  |  |  |  | 1 |
| miR-134-5p |  |  |  | △ |  |  |  |  |  |  |  |  |  |  |  |  |  |  |  |  |  |  |  | 1 |
| miR-139-5p |  |  |  | △ |  |  |  |  |  |  |  |  |  |  |  |  |  |  |  |  |  |  |  | 1 |
| miR-140-3p |  |  |  | △ |  |  |  |  |  |  |  |  |  |  |  |  |  |  |  |  |  |  |  | 1 |
| miR-142-5p |  |  |  | △ |  |  |  |  |  |  |  |  |  |  |  |  |  |  |  |  |  |  |  | 1 |
| miR-143-3p |  |  |  | ↑△ |  |  |  |  |  |  |  |  |  |  |  |  |  |  |  |  |  |  |  | 1 |
| miR-145-3p |  |  |  | ↑△ |  |  |  |  |  |  |  |  |  |  |  |  |  |  |  |  |  |  |  | 1 |
| miR-148b-3p |  |  |  | △ |  |  |  |  |  |  |  |  |  |  |  |  |  |  |  |  |  |  |  | 1 |
| miR-151a-3p |  |  |  | △ |  |  |  |  |  |  |  |  |  |  |  |  |  |  |  |  |  |  |  | 1 |
| miR-15a-5p |  |  |  | △ |  |  |  |  |  |  |  |  |  |  |  |  |  |  |  |  |  |  |  | 1 |
| miR-15b-3p |  |  |  | △ |  |  |  |  |  |  |  |  |  |  |  |  |  |  |  |  |  |  |  | 1 |
| miR-16-2-3p |  |  |  | △ |  |  |  |  |  |  |  |  |  |  |  |  |  |  |  |  |  |  |  | 1 |
| miR-16-5p |  |  |  | △ |  |  |  |  |  |  |  |  |  |  |  |  |  |  |  |  |  |  |  | 1 |
| miR-17-5p |  |  |  | △ |  |  |  |  |  |  |  |  |  |  |  |  |  |  |  |  |  |  |  | 1 |
| miR-181a-2-3p |  |  |  | △ |  |  |  |  |  |  |  |  |  |  |  |  |  |  |  |  |  |  |  | 1 |
| miR-182-5p |  |  |  | △ |  |  |  |  |  |  |  |  |  |  |  |  |  |  |  |  |  |  |  | 1 |
| miR-183-5p |  |  |  | △ |  |  |  |  |  |  |  |  |  |  |  |  |  |  |  |  |  |  |  | 1 |
| miR-185-3p |  |  |  | △ |  |  |  |  |  |  |  |  |  |  |  |  |  |  |  |  |  |  |  | 1 |
| miR-185-5p |  |  |  | △ |  |  |  |  |  |  |  |  |  |  |  |  |  |  |  |  |  |  |  | 1 |
| miR-186-5p |  |  |  | △ |  |  |  |  |  |  |  |  |  |  |  |  |  |  |  |  |  |  |  | 1 |
| miR-18a-3p |  |  |  | △ |  |  |  |  |  |  |  |  |  |  |  |  |  |  |  |  |  |  |  | 1 |
| miR-1908-5p |  |  |  | △ |  |  |  |  |  |  |  |  |  |  |  |  |  |  |  |  |  |  |  | 1 |
|  |  |  |  |  |  |  |  |  |  |  |  |  |  |  |  |  |  |  |  |  |  |  |  |  |
| **Additional file 2 continue** | | | | | | | | | | | | | | | | | | | | | | | | |
|  | **Ref.** | | | | | | | | | | | | | | | | | | | | | | |  |
| **RNA** | **Chen, 2022** | **Flammang, 2020** | **Goto, 2018** | **Guo, 2021** | **Hu, 2017** | **Kim, 2021** | **Kitagawa, 2019** | **Lai, 2017** | **Madhavan, 2015** | **Pu, 2020** | **Qin, 2021** | **Reese, 2020** | **Shao, 2021** | **Takahashi, 2019** | **Verel-Y,2021** | **Wang L, 2021** | **Wang, 2021** | **Wu, 2020** | **Wu, 2021** | **Xiao, 2021** | **Xu, 2017** | **Yu, 2019** | **Zhou, 2020** | **number of studies** |
| miR-192-5p |  | –○ |  |  |  |  |  |  |  |  |  |  |  |  |  |  |  |  |  |  |  |  |  | 1 |
| miR-196a |  |  |  |  |  |  |  |  |  |  |  |  |  |  |  |  |  |  |  |  | ↑○ |  |  | 1 |
| miR-196b |  |  |  |  |  |  |  |  |  |  |  |  |  |  |  |  |  |  |  |  | –○ |  |  | 1 |
| miR-197-3p |  |  |  | △ |  |  |  |  |  |  |  |  |  |  |  |  |  |  |  |  |  |  |  | 1 |
| miR-199a-3p |  |  |  | △ |  |  |  |  |  |  |  |  |  |  |  |  |  |  |  |  |  |  |  | 1 |
| miR-199b-3p |  |  |  | ↑△ |  |  |  |  |  |  |  |  |  |  |  |  |  |  |  |  |  |  |  | 1 |
| miR-19b-3p |  |  |  |  |  |  |  |  |  |  |  |  |  |  |  | ↓○ |  |  |  |  |  |  |  | 1 |
| miR-200b |  |  |  |  |  |  |  |  |  |  |  | ↑△ |  |  |  |  |  |  |  |  |  |  |  | 1 |
| miR-200c |  |  |  |  |  |  |  |  |  |  |  | ↑△ |  |  |  |  |  |  |  |  |  |  |  | 1 |
| miR-206 |  |  |  | △ |  |  |  |  |  |  |  |  |  |  |  |  |  |  |  |  |  |  |  | 1 |
| miR-20b-5p |  |  |  | △ |  |  |  |  |  |  |  |  |  |  |  |  |  |  |  |  |  |  |  | 1 |
| miR-210 |  |  |  |  |  |  |  |  |  |  |  |  |  |  |  |  |  | ↑△ |  |  |  |  |  | 1 |
| miR-210-3p |  |  |  | △ |  |  |  |  |  |  |  |  |  |  |  |  |  |  |  |  |  |  |  | 1 |
| miR-2110 |  |  |  | △ |  |  |  |  |  |  |  |  |  |  |  |  |  |  |  |  |  |  |  | 1 |
| miR-223-3p |  |  |  | ↑△ |  |  |  |  |  |  |  |  |  |  |  |  |  |  |  |  |  |  |  | 1 |
| miR-224-5p |  |  |  | △ |  |  |  |  |  |  |  |  |  |  |  |  |  |  |  |  |  |  |  | 1 |
| miR-22-5p |  |  |  | △ |  |  |  |  |  |  |  |  |  |  |  |  |  |  |  |  |  |  |  | 1 |
| miR-23a-3p |  |  |  | △ |  |  |  |  |  |  |  |  |  |  |  |  |  |  |  |  |  |  |  | 1 |
| miR-24-2-5p |  |  |  | △ |  |  |  |  |  |  |  |  |  |  |  |  |  |  |  |  |  |  |  | 1 |
| miR-24-3p |  |  |  | △ |  |  |  |  |  |  |  |  |  |  |  |  |  |  |  |  |  |  |  | 1 |
| miR-25-3p |  |  |  | △ |  |  |  |  |  |  |  |  |  |  |  |  |  |  |  |  |  |  |  | 1 |
| miR-25-5p |  |  |  | △ |  |  |  |  |  |  |  |  |  |  |  |  |  |  |  |  |  |  |  | 1 |
| miR-26b-5p |  |  |  | △ |  |  |  |  |  |  |  |  |  |  |  |  |  |  |  |  |  |  |  | 1 |
| miR-27a-3p |  |  |  | △ |  |  |  |  |  |  |  |  |  |  |  |  |  |  |  |  |  |  |  | 1 |
| miR-27b-3p |  |  |  | ↑△ |  |  |  |  |  |  |  |  |  |  |  |  |  |  |  |  |  |  |  | 1 |
| miR-29a-3p |  |  |  | △ |  |  |  |  |  |  |  |  |  |  |  |  |  |  |  |  |  |  |  | 1 |
|  |  |  |  |  |  |  |  |  |  |  |  |  |  |  |  |  |  |  |  |  |  |  |  |  |
| **Additional file 2 continue** | | | | | | | | | | | | | | | | | | | | | | | | |
|  | **Ref.** | | | | | | | | | | | | | | | | | | | | | | |  |
| **RNA** | **Chen, 2022** | **Flammang, 2020** | **Goto, 2018** | **Guo, 2021** | **Hu, 2017** | **Kim, 2021** | **Kitagawa, 2019** | **Lai, 2017** | **Madhavan, 2015** | **Pu, 2020** | **Qin, 2021** | **Reese, 2020** | **Shao, 2021** | **Takahashi, 2019** | **Verel-Y,2021** | **Wang L, 2021** | **Wang, 2021** | **Wu, 2020** | **Wu, 2021** | **Xiao, 2021** | **Xu, 2017** | **Yu, 2019** | **Zhou, 2020** | **number of studies** |
| miR-30a-5p |  |  |  | △ |  |  |  |  |  |  |  |  |  |  |  |  |  |  |  |  |  |  |  | 1 |
| miR-30c |  |  |  |  |  |  |  | ↑○ |  |  |  |  |  |  |  |  |  |  |  |  |  |  |  | 1 |
| miR-30d-5p |  |  |  | △ |  |  |  |  |  |  |  |  |  |  |  |  |  |  |  |  |  |  |  | 1 |
| miR-30e-5p |  |  |  | △ |  |  |  |  |  |  |  |  |  |  |  |  |  |  |  |  |  |  |  | 1 |
| miR-3158-3p |  |  |  | ↓△ |  |  |  |  |  |  |  |  |  |  |  |  |  |  |  |  |  |  |  | 1 |
| miR-320a |  |  |  | △ |  |  |  |  |  |  |  |  |  |  |  |  |  |  |  |  |  |  |  | 1 |
| miR-32-5p |  |  |  | △ |  |  |  |  |  |  |  |  |  |  |  |  |  |  |  |  |  |  |  | 1 |
| miR-335-5p |  |  |  | △ |  |  |  |  |  |  |  |  |  |  |  |  |  |  |  |  |  |  |  | 1 |
| miR-338-5p |  |  |  | △ |  |  |  |  |  |  |  |  |  |  |  |  |  |  |  |  |  |  |  | 1 |
| miR-339-3p |  |  |  | △ |  |  |  |  |  |  |  |  |  |  |  |  |  |  |  |  |  |  |  | 1 |
| miR-342-5p |  |  |  | △ |  |  |  |  |  |  |  |  |  |  |  |  |  |  |  |  |  |  |  | 1 |
| miR-3605-3p |  |  |  | △ |  |  |  |  |  |  |  |  |  |  |  |  |  |  |  |  |  |  |  | 1 |
| miR-3613-5p |  |  |  | △ |  |  |  |  |  |  |  |  |  |  |  |  |  |  |  |  |  |  |  | 1 |
| miR-3615 |  |  |  | △ |  |  |  |  |  |  |  |  |  |  |  |  |  |  |  |  |  |  |  | 1 |
| miR-361-5p |  |  |  | △ |  |  |  |  |  |  |  |  |  |  |  |  |  |  |  |  |  |  |  | 1 |
| miR-363-3p |  |  |  | △ |  |  |  |  |  |  |  |  |  |  |  |  |  |  |  |  |  |  |  | 1 |
| miR-363-5p |  |  |  | △ |  |  |  |  |  |  |  |  |  |  |  |  |  |  |  |  |  |  |  | 1 |
| miR-382-5p |  |  |  | △ |  |  |  |  |  |  |  |  |  |  |  |  |  |  |  |  |  |  |  | 1 |
| miR-3976 |  |  |  |  |  |  |  |  | △ |  |  |  |  |  |  |  |  |  |  |  |  |  |  | 1 |
| miR-421 |  |  |  | △ |  |  |  |  |  |  |  |  |  |  |  |  |  |  |  |  |  |  |  | 1 |
| miR-423-5p |  |  |  | △ |  |  |  |  |  |  |  |  |  |  |  |  |  |  |  |  |  |  |  | 1 |
| miR-425-5p |  |  |  | △ |  |  |  |  |  |  |  |  |  |  |  |  |  |  |  |  |  |  |  | 1 |
| miR-429 |  |  |  |  |  | △ |  |  |  |  |  |  |  |  |  |  |  |  |  |  |  |  |  | 1 |
| miR-4306 |  |  |  |  |  |  |  |  | △ |  |  |  |  |  |  |  |  |  |  |  |  |  |  | 1 |
| miR-4326 |  |  |  | △ |  |  |  |  |  |  |  |  |  |  |  |  |  |  |  |  |  |  |  | 1 |
| miR-4433b-3p |  |  |  | △ |  |  |  |  |  |  |  |  |  |  |  |  |  |  |  |  |  |  |  | 1 |
|  |  |  |  |  |  |  |  |  |  |  |  |  |  |  |  |  |  |  |  |  |  |  |  |  |
| **Additional file 2 continue** | | | | | | | | | | | | | | | | | | | | | | | | |
|  | **Ref.** | | | | | | | | | | | | | | | | | | | | | | |  |
| **RNA** | **Chen, 2022** | **Flammang, 2020** | **Goto, 2018** | **Guo, 2021** | **Hu, 2017** | **Kim, 2021** | **Kitagawa, 2019** | **Lai, 2017** | **Madhavan, 2015** | **Pu, 2020** | **Qin, 2021** | **Reese, 2020** | **Shao, 2021** | **Takahashi, 2019** | **Verel-Y,2021** | **Wang L, 2021** | **Wang, 2021** | **Wu, 2020** | **Wu, 2021** | **Xiao, 2021** | **Xu, 2017** | **Yu, 2019** | **Zhou, 2020** | **number of studies** |
| miR-450b-5p |  |  |  | △ |  |  |  |  |  |  |  |  |  |  |  |  |  |  |  |  |  |  |  | 1 |
| miR-451 |  |  |  |  |  |  |  |  |  |  |  |  |  |  | △ |  |  |  |  |  |  |  |  | 1 |
| miR-4644 |  |  |  |  |  |  |  |  | △ |  |  |  |  |  |  |  |  |  |  |  |  |  |  | 1 |
| miR-4732-3p |  |  |  | ↑△ |  |  |  |  |  |  |  |  |  |  |  |  |  |  |  |  |  |  |  | 1 |
| miR-4732-5p |  |  |  | △ |  |  |  |  |  |  |  |  |  |  |  |  |  |  |  |  |  |  |  | 1 |
| miR-483-3p |  |  |  |  |  |  |  |  |  |  |  |  | ↑○ |  |  |  |  |  |  |  |  |  |  | 1 |
| miR-484 |  |  |  | △ |  |  |  |  |  |  |  |  |  |  |  |  |  |  |  |  |  |  |  | 1 |
| miR-485-5p |  |  |  | △ |  |  |  |  |  |  |  |  |  |  |  |  |  |  |  |  |  |  |  | 1 |
| miR-486-3p |  |  |  | ↓△ |  |  |  |  |  |  |  |  |  |  |  |  |  |  |  |  |  |  |  | 1 |
| miR-486-5p |  |  |  | △ |  |  |  |  |  |  |  |  |  |  |  |  |  |  |  |  |  |  |  | 1 |
| miR-499a-5p |  |  |  | △ |  |  |  |  |  |  |  |  |  |  |  |  |  |  |  |  |  |  |  | 1 |
| miR-501-3p |  |  |  | △ |  |  |  |  |  |  |  |  |  |  |  |  |  |  |  |  |  |  |  | 1 |
| miR-503-5p |  |  |  | △ |  |  |  |  |  |  |  |  |  |  |  |  |  |  |  |  |  |  |  | 1 |
| miR-505-5p |  |  |  | △ |  |  |  |  |  |  |  |  |  |  |  |  |  |  |  |  |  |  |  | 1 |
| miR-511-5p |  |  |  | ↑△ |  |  |  |  |  |  |  |  |  |  |  |  |  |  |  |  |  |  |  | 1 |
| miR-532-5p |  |  |  | △ |  |  |  |  |  |  |  |  |  |  |  |  |  |  |  |  |  |  |  | 1 |
| miR-543 |  |  |  | △ |  |  |  |  |  |  |  |  |  |  |  |  |  |  |  |  |  |  |  | 1 |
| miR-550a-3-5p |  |  |  | △ |  |  |  |  |  |  |  |  |  |  |  |  |  |  |  |  |  |  |  | 1 |
| miR-550a-5p |  |  |  | △ |  |  |  |  |  |  |  |  |  |  |  |  |  |  |  |  |  |  |  | 1 |
| miR-584-5p |  |  |  | △ |  |  |  |  |  |  |  |  |  |  |  |  |  |  |  |  |  |  |  | 1 |
| miR-628-3p |  |  |  | △ |  |  |  |  |  |  |  |  |  |  |  |  |  |  |  |  |  |  |  | 1 |
| miR-629-5p |  |  |  | △ |  |  |  |  |  |  |  |  |  |  |  |  |  |  |  |  |  |  |  | 1 |
| miR-652-3p |  |  |  | △ |  |  |  |  |  |  |  |  |  |  |  |  |  |  |  |  |  |  |  | 1 |
| miR-660-5p |  |  |  | △ |  |  |  |  |  |  |  |  |  |  |  |  |  |  |  |  |  |  |  | 1 |
| miR663AHG |  |  |  |  |  |  |  |  |  |  |  |  |  |  |  |  |  |  | △ |  |  |  |  | 1 |
| miR-6842-3p |  |  |  | △ |  |  |  |  |  |  |  |  |  |  |  |  |  |  |  |  |  |  |  | 1 |
|  |  |  |  |  |  |  |  |  |  |  |  |  |  |  |  |  |  |  |  |  |  |  |  |  |
| **Additional file 2 continue** | | | | | | | | | | | | | | | | | | | | | | | | |
|  | **Ref.** | | | | | | | | | | | | | | | | | | | | | | |  |
| **RNA** | **Chen, 2022** | **Flammang, 2020** | **Goto, 2018** | **Guo, 2021** | **Hu, 2017** | **Kim, 2021** | **Kitagawa, 2019** | **Lai, 2017** | **Madhavan, 2015** | **Pu, 2020** | **Qin, 2021** | **Reese, 2020** | **Shao, 2021** | **Takahashi, 2019** | **Verel-Y,2021** | **Wang L, 2021** | **Wang, 2021** | **Wu, 2020** | **Wu, 2021** | **Xiao, 2021** | **Xu, 2017** | **Yu, 2019** | **Zhou, 2020** | **number of studies** |
| miR-720 |  |  |  |  |  |  |  |  |  |  |  |  |  |  | △ |  |  |  |  |  |  |  |  | 1 |
| miR-7-5p |  |  |  | △ |  |  |  |  |  |  |  |  |  |  |  |  |  |  |  |  |  |  |  | 1 |
| miR-760 |  |  |  | △ |  |  |  |  |  |  |  |  |  |  |  |  |  |  |  |  |  |  |  | 1 |
| miR-7706 |  |  |  | ↓△ |  |  |  |  |  |  |  |  |  |  |  |  |  |  |  |  |  |  |  | 1 |
| miR-7976 |  |  |  | △ |  |  |  |  |  |  |  |  |  |  |  |  |  |  |  |  |  |  |  | 1 |
| miR-92a-3p |  |  |  | △ |  |  |  |  |  |  |  |  |  |  |  |  |  |  |  |  |  |  |  | 1 |
| miR-92b-3p |  |  |  | △ |  |  |  |  |  |  |  |  |  |  |  |  |  |  |  |  |  |  |  | 1 |
| miR-92b-5p |  |  |  | △ |  |  |  |  |  |  |  |  |  |  |  |  |  |  |  |  |  |  |  | 1 |
| miR-93-5p |  |  |  | △ |  |  |  |  |  |  |  |  |  |  |  |  |  |  |  |  |  |  |  | 1 |
| miR-941 |  |  |  | △ |  |  |  |  |  |  |  |  |  |  |  |  |  |  |  |  |  |  |  | 1 |
| miR-942-5p |  |  |  | △ |  |  |  |  |  |  |  |  |  |  |  |  |  |  |  |  |  |  |  | 1 |
| miR-95-3p |  |  |  | ↑△ |  |  |  |  |  |  |  |  |  |  |  |  |  |  |  |  |  |  |  | 1 |
| miR-99a-5p |  |  |  | ↑223△ |  |  |  |  |  |  |  |  |  |  |  |  |  |  |  |  |  |  |  | 1 |
| miR-99b-5p |  |  |  | ↑△ |  |  |  |  |  |  |  |  |  |  |  |  |  |  |  |  |  |  |  | 1 |
| miR-let7a |  |  |  |  |  |  |  | ↓○ |  |  |  |  |  |  |  |  |  |  |  |  |  |  |  | 1 |
| MORF4L1 |  |  |  |  |  |  |  |  |  |  | △ |  |  |  |  |  |  |  |  |  |  |  |  | 1 |
| NEUROD2 |  |  |  |  |  |  |  |  |  |  |  |  |  |  |  |  |  |  | △ |  |  |  |  | 1 |
| RN7SL1 |  |  |  |  |  |  |  |  |  |  |  |  |  |  |  |  |  |  | △ |  |  |  |  | 1 |
| SETD3 |  |  |  |  |  |  |  |  |  |  | △ |  |  |  |  |  |  |  |  |  |  |  |  | 1 |
| SNORA14B |  |  |  |  |  |  | ↑○ |  |  |  |  |  |  |  |  |  |  |  |  |  |  |  |  | 1 |
| SNORA18 |  |  |  |  |  |  | ↑○ |  |  |  |  |  |  |  |  |  |  |  |  |  |  |  |  | 1 |
| SNORA25 |  |  |  |  |  |  | ↑○ |  |  |  |  |  |  |  |  |  |  |  |  |  |  |  |  | 1 |
| SNORA74A |  |  |  |  |  |  | ↑○ |  |  |  |  |  |  |  |  |  |  |  |  |  |  |  |  | 1 |
| SNORD22 |  |  |  |  |  |  | ↑○ |  |  |  |  |  |  |  |  |  |  |  |  |  |  |  |  | 1 |
| TALDO1 |  |  |  |  |  |  |  |  |  |  | △ |  |  |  |  |  |  |  |  |  |  |  |  | 1 |
| TIMP1 |  |  |  |  |  |  |  |  |  |  |  |  |  |  |  |  |  |  |  |  |  | △ |  | 1 |
|  |  |  |  |  |  |  |  |  |  |  |  |  |  |  |  |  |  |  |  |  |  |  |  |  |
|  |  |  |  |  |  |  |  |  |  |  |  |  |  |  |  |  |  |  |  |  |  |  |  |  |
| **Additional file 2 continue** | | | | | | | | | | | | | | | | | | | | | | | | |
|  | **Ref.** | | | | | | | | | | | | | | | | | | | | | | |  |
| **RNA** | **Chen, 2022** | **Flammang, 2020** | **Goto, 2018** | **Guo, 2021** | **Hu, 2017** | **Kim, 2021** | **Kitagawa, 2019** | **Lai, 2017** | **Madhavan, 2015** | **Pu, 2020** | **Qin, 2021** | **Reese, 2020** | **Shao, 2021** | **Takahashi, 2019** | **Verel-Y,2021** | **Wang L, 2021** | **Wang, 2021** | **Wu, 2020** | **Wu, 2021** | **Xiao, 2021** | **Xu, 2017** | **Yu, 2019** | **Zhou, 2020** | **number of studies** |
| TUBA1B |  |  |  |  |  |  |  |  |  |  | △ |  |  |  |  |  |  |  |  |  |  |  |  | 1 |
| Vav3 |  |  |  |  |  |  | ↑○ |  |  |  |  |  |  |  |  |  |  |  |  |  |  |  |  | 1 |
| WASF2 |  |  |  |  |  |  | ↑○ |  |  |  |  |  |  |  |  |  |  |  |  |  |  |  |  | 1 |
| MARCH2 |  |  |  |  |  |  |  |  |  |  |  |  |  |  |  |  |  |  |  |  |  | △ |  | 1 |

○ represents RNAs which have only been analyzed individually and not as part of a miRNA panel; △ represents RNAs which are part of a panel; ↑ represents up-regulation; ↓ represents down-regulation.
