# Supplementary material for: Extracellular vesicle biomarkers for pancreatic cancer diagnosis: a systematic review and meta-analysis
Source: BMC Cancer. 2022 May 23;22:573. doi: 10.1186/s12885-022-09463-x (PMC9125932; doi:10.1186/s12885-022-09463-x)
Supplement: Supplementary file 3 — Additional file 3. Summary of studies reporting significant associations of proteins in pancreatic cancer. [file 12885_2022_9463_MOESM3_ESM.docx]

| **Additional file 3. Summary of studies reporting significant associations of proteins in pancreatic cancer** | | | | | | | | | | | | | | | | | | | | | |
| --- | --- | --- | --- | --- | --- | --- | --- | --- | --- | --- | --- | --- | --- | --- | --- | --- | --- | --- | --- | --- | --- |
|  | **Ref.** | | | | | | | | | | | | | | | | | | | |  |
| **Protein** | **Buscail, 2019** | **Fahrmann,2020** | **Jin, 2018** | **Kim, 2021** | **Lewis, 2018** | **Li, 2018** | **Li, 2021** | **Liang, 2017** | **Lux, 2019** | **Madhavan, 2015** | **Melo, 2015** | **Moutinho-R, 2021** | **Rodrigues, 2019** | **Verel-Y,2021** | **Wei, 2020** | **Xiao, 2019** | **Yang, 2017** | **Yang, 2021** | **Zheng, 2022** | **Zhou, 2020** | **number of studies** |
| GPC1 | ○ |  |  | △ | △ | ○ | △ |  |  |  | ○ | ○ |  |  |  | △ | △ |  |  |  | 9 |
| EphA2 |  | ○ |  |  |  |  | △ | ○ |  |  |  |  | △ |  | ○ |  |  |  |  | △ | 6 |
| EPCAM |  |  |  |  |  |  | △ |  |  | △ |  |  | △ |  |  |  | △ |  |  |  | 4 |
| EGFR |  |  |  |  |  | ○ | △ |  |  |  |  |  |  |  |  |  | △ |  |  |  | 3 |
| CLEC11A |  | ○ |  |  |  |  |  |  |  |  |  |  |  |  |  |  |  |  |  |  | 1 |
| ABL2 |  | ○ |  |  |  |  |  |  |  |  |  |  |  |  |  |  |  |  |  |  | 1 |
| ACVRL1 |  | ○ |  |  |  |  |  |  |  |  |  |  |  |  |  |  |  |  |  |  | 1 |
| ADAM8 |  |  |  |  |  |  |  |  |  |  |  |  |  | △ |  |  |  |  |  |  | 1 |
| ADAM9 |  | ○ |  |  |  |  |  |  |  |  |  |  |  |  |  |  |  |  |  |  | 1 |
| AFP |  | ○ |  |  |  |  |  |  |  |  |  |  |  |  |  |  |  |  |  |  | 1 |
| AIMP1 |  | ○ |  |  |  |  |  |  |  |  |  |  |  |  |  |  |  |  |  |  | 1 |
| AIP |  | ○ |  |  |  |  |  |  |  |  |  |  |  |  |  |  |  |  |  |  | 1 |
| ALIX |  |  |  |  |  |  |  |  |  |  |  |  |  |  |  |  |  | ○ |  |  | 1 |
| ANXA1 |  | ○ |  |  |  |  |  |  |  |  |  |  |  |  |  |  |  |  |  |  | 1 |
| APOD |  | ○ |  |  |  |  |  |  |  |  |  |  |  |  |  |  |  |  |  |  | 1 |
| ASGR1 |  | ○ |  |  |  |  |  |  |  |  |  |  |  |  |  |  |  |  |  |  | 1 |
|  |  |  |  |  |  |  |  |  |  |  |  |  |  |  |  |  |  |  |  |  |  |
| **Additional file 3 continue** | | | | | | | | | | | | | | | | | | | | | |
|  | **Ref.** | | | | | | | | | | | | | | | | | | | |  |
| **Protein** | **Buscail, 2019** | **Fahrmann,2020** | **Jin, 2018** | **Kim, 2021** | **Lewis, 2018** | **Li, 2018** | **Li, 2021** | **Liang, 2017** | **Lux, 2019** | **Madhavan, 2015** | **Melo, 2015** | **Moutinho-R, 2021** | **Rodrigues, 2019** | **Verel-Y,2021** | **Wei, 2020** | **Xiao, 2019** | **Yang, 2017** | **Yang, 2021** | **Zheng, 2022** | **Zhou, 2020** | **number of studies** |
| B2M |  | ○ |  |  |  |  |  |  |  |  |  |  |  |  |  |  |  |  |  |  | 1 |
| B7-H3 |  |  |  |  |  |  |  |  |  |  |  |  |  |  |  |  | ○ |  |  |  | 1 |
| BCL2 |  | ○ |  |  |  |  |  |  |  |  |  |  |  |  |  |  |  |  |  |  | 1 |
| BCL2L1 |  | ○ |  |  |  |  |  |  |  |  |  |  |  |  |  |  |  |  |  |  | 1 |
| BCL2L2 |  | ○ |  |  |  |  |  |  |  |  |  |  |  |  |  |  |  |  |  |  | 1 |
| BIRC5 |  | ○ |  |  |  |  |  |  |  |  |  |  |  |  |  |  |  |  |  |  | 1 |
| BMPR2 |  | ○ |  |  |  |  |  |  |  |  |  |  |  |  |  |  |  |  |  |  | 1 |
| C1QBP |  | ○ |  |  |  |  |  |  |  |  |  |  |  |  |  |  |  |  |  |  | 1 |
| C5 C6 |  | ○ |  |  |  |  |  |  |  |  |  |  |  |  |  |  |  |  |  |  | 1 |
| CA10 |  | ○ |  |  |  |  |  |  |  |  |  |  |  |  |  |  |  |  |  |  | 1 |
| CA3 |  | ○ |  |  |  |  |  |  |  |  |  |  |  |  |  |  |  |  |  |  | 1 |
| CAMK2A |  | ○ |  |  |  |  |  |  |  |  |  |  |  |  |  |  |  |  |  |  | 1 |
| CCL17 |  | ○ |  |  |  |  |  |  |  |  |  |  |  |  |  |  |  |  |  |  | 1 |
| CCL22 |  | ○ |  |  |  |  |  |  |  |  |  |  |  |  |  |  |  |  |  |  | 1 |
| CCL28 |  | ○ |  |  |  |  |  |  |  |  |  |  |  |  |  |  |  |  |  |  | 1 |
| CCL3L1 |  | ○ |  |  |  |  |  |  |  |  |  |  |  |  |  |  |  |  |  |  | 1 |
| CD104 |  |  |  |  |  |  |  |  |  | △ |  |  |  |  |  |  |  |  |  |  | 1 |
|  |  |  |  |  |  |  |  |  |  |  |  |  |  |  |  |  |  |  |  |  |  |
| **Additional file 3 continue** | | | | | | | | | | | | | | | | | | | | | |
|  | **Ref.** | | | | | | | | | | | | | | | | | | | |  |
| **Protein** | **Buscail, 2019** | **Fahrmann,2020** | **Jin, 2018** | **Kim, 2021** | **Lewis, 2018** | **Li, 2018** | **Li, 2021** | **Liang, 2017** | **Lux, 2019** | **Madhavan, 2015** | **Melo, 2015** | **Moutinho-R, 2021** | **Rodrigues, 2019** | **Verel-Y,2021** | **Wei, 2020** | **Xiao, 2019** | **Yang, 2017** | **Yang, 2021** | **Zheng, 2022** | **Zhou, 2020** | **number of studies** |
| CD109 |  | ○ |  |  |  |  |  |  |  |  |  |  |  |  |  |  |  |  |  |  | 1 |
| CD244 |  | ○ |  |  |  |  |  |  |  |  |  |  |  |  |  |  |  |  |  |  | 1 |
| CD300C |  | ○ |  |  |  |  |  |  |  |  |  |  |  |  |  |  |  |  |  |  | 1 |
| CD4 |  | ○ |  |  |  |  |  |  |  |  |  |  |  |  |  |  |  |  |  |  | 1 |
| CD44v6 |  |  |  |  |  |  |  |  |  | △ |  |  |  |  |  |  |  |  |  |  | 1 |
| CD47 |  | ○ |  |  |  |  |  |  |  |  |  |  |  |  |  |  |  |  |  |  | 1 |
| CD63 |  |  |  |  | △ |  |  |  |  |  |  |  |  |  |  |  |  |  |  |  | 1 |
| CD82 |  |  |  |  |  |  |  |  |  |  |  |  |  |  |  | △ |  |  |  |  | 1 |
| CDH3 |  | ○ |  |  |  |  |  |  |  |  |  |  |  |  |  |  |  |  |  |  | 1 |
| CDK2 CCNA2 | | ○ |  |  |  |  |  |  |  |  |  |  |  |  |  |  |  |  |  |  | 1 |
| CEBPB |  | ○ |  |  |  |  |  |  |  |  |  |  |  |  |  |  |  |  |  |  | 1 |
| CHEK2 |  | ○ |  |  |  |  |  |  |  |  |  |  |  |  |  |  |  |  |  |  | 1 |
| CKM |  | ○ |  |  |  |  |  |  |  |  |  |  |  |  |  |  |  |  |  |  | 1 |
| c-Met |  |  |  |  |  |  |  |  | ○ |  |  |  |  |  |  |  |  |  |  |  | 1 |
| CRLF1 CLCF1 | | ○ |  |  |  |  |  |  |  |  |  |  |  |  |  |  |  |  |  |  | 1 |
| CRLF2 |  | ○ |  |  |  |  |  |  |  |  |  |  |  |  |  |  |  |  |  |  | 1 |
| CSNK2A1 |  | ○ |  |  |  |  |  |  |  |  |  |  |  |  |  |  |  |  |  |  | 1 |
|  |  |  |  |  |  |  |  |  |  |  |  |  |  |  |  |  |  |  |  |  |  |
| **Additional file 3 continue** | | | | | | | | | | | | | | | | | | | | | |
|  | **Ref.** | | | | | | | | | | | | | | | | | | | |  |
| **Protein** | **Buscail, 2019** | **Fahrmann,2020** | **Jin, 2018** | **Kim, 2021** | **Lewis, 2018** | **Li, 2018** | **Li, 2021** | **Liang, 2017** | **Lux, 2019** | **Madhavan, 2015** | **Melo, 2015** | **Moutinho-R, 2021** | **Rodrigues, 2019** | **Verel-Y,2021** | **Wei, 2020** | **Xiao, 2019** | **Yang, 2017** | **Yang, 2021** | **Zheng, 2022** | **Zhou, 2020** | **number of studies** |
| CSRP3 |  | ○ |  |  |  |  |  |  |  |  |  |  |  |  |  |  |  |  |  |  | 1 |
| CTF1 |  | ○ |  |  |  |  |  |  |  |  |  |  |  |  |  |  |  |  |  |  | 1 |
| CXCL1 |  | ○ |  |  |  |  |  |  |  |  |  |  |  |  |  |  |  |  |  |  | 1 |
| CXCL5 |  | ○ |  |  |  |  |  |  |  |  |  |  |  |  |  |  |  |  |  |  | 1 |
| CXCL8 |  | ○ |  |  |  |  |  |  |  |  |  |  |  |  |  |  |  |  |  |  | 1 |
| DDR1 |  | ○ |  |  |  |  |  |  |  |  |  |  |  |  |  |  |  |  |  |  | 1 |
| DHH |  | ○ |  |  |  |  |  |  |  |  |  |  |  |  |  |  |  |  |  |  | 1 |
| DYNLL1 |  | ○ |  |  |  |  |  |  |  |  |  |  |  |  |  |  |  |  |  |  | 1 |
| DYNLRB1 |  | ○ |  |  |  |  |  |  |  |  |  |  |  |  |  |  |  |  |  |  | 1 |
| EDA |  | ○ |  |  |  |  |  |  |  |  |  |  |  |  |  |  |  |  |  |  | 1 |
| ENTPD5 |  | ○ |  |  |  |  |  |  |  |  |  |  |  |  |  |  |  |  |  |  | 1 |
| EPHA1 |  | ○ |  |  |  |  |  |  |  |  |  |  |  |  |  |  |  |  |  |  | 1 |
| EPHA5 |  | ○ |  |  |  |  |  |  |  |  |  |  |  |  |  |  |  |  |  |  | 1 |
| ERP29 |  | ○ |  |  |  |  |  |  |  |  |  |  |  |  |  |  |  |  |  |  | 1 |
| ETHE1 |  | ○ |  |  |  |  |  |  |  |  |  |  |  |  |  |  |  |  |  |  | 1 |
| F3 |  | ○ |  |  |  |  |  |  |  |  |  |  |  |  |  |  |  |  |  |  | 1 |
| FABP1 |  | ○ |  |  |  |  |  |  |  |  |  |  |  |  |  |  |  |  |  |  | 1 |
|  |  |  |  |  |  |  |  |  |  |  |  |  |  |  |  |  |  |  |  |  |  |
| **Additional file 3 continue** | | | | | | | | | | | | | | | | | | | | | |
|  | **Ref.** | | | | | | | | | | | | | | | | | | | |  |
| **Protein** | **Buscail, 2019** | **Fahrmann,2020** | **Jin, 2018** | **Kim, 2021** | **Lewis, 2018** | **Li, 2018** | **Li, 2021** | **Liang, 2017** | **Lux, 2019** | **Madhavan, 2015** | **Melo, 2015** | **Moutinho-R, 2021** | **Rodrigues, 2019** | **Verel-Y,2021** | **Wei, 2020** | **Xiao, 2019** | **Yang, 2017** | **Yang, 2021** | **Zheng, 2022** | **Zhou, 2020** | **number of studies** |
| FABP5 |  | ○ |  |  |  |  |  |  |  |  |  |  |  |  |  |  |  |  |  |  | 1 |
| FAM107A |  | ○ |  |  |  |  |  |  |  |  |  |  |  |  |  |  |  |  |  |  | 1 |
| FAS |  | ○ |  |  |  |  |  |  |  |  |  |  |  |  |  |  |  |  |  |  | 1 |
| FGF2 |  | ○ |  |  |  |  |  |  |  |  |  |  |  |  |  |  |  |  |  |  | 1 |
| FGF5 |  | ○ |  |  |  |  |  |  |  |  |  |  |  |  |  |  |  |  |  |  | 1 |
| FGFR4 |  | ○ |  |  |  |  |  |  |  |  |  |  |  |  |  |  |  |  |  |  | 1 |
| FGG |  |  |  |  |  |  |  |  |  |  |  |  |  |  |  |  |  |  | ○ |  | 1 |
| FLRT3 |  | ○ |  |  |  |  |  |  |  |  |  |  |  |  |  |  |  |  |  |  | 1 |
| FLT3 |  | ○ |  |  |  |  |  |  |  |  |  |  |  |  |  |  |  |  |  |  | 1 |
| FLT3LG |  | ○ |  |  |  |  |  |  |  |  |  |  |  |  |  |  |  |  |  |  | 1 |
| GCKR |  | ○ |  |  |  |  |  |  |  |  |  |  |  |  |  |  |  |  |  |  | 1 |
| GDF11 |  | ○ |  |  |  |  |  |  |  |  |  |  |  |  |  |  |  |  |  |  | 1 |
| GDF2 |  | ○ |  |  |  |  |  |  |  |  |  |  |  |  |  |  |  |  |  |  | 1 |
| GDF5 |  | ○ |  |  |  |  |  |  |  |  |  |  |  |  |  |  |  |  |  |  | 1 |
| GDF9 |  | ○ |  |  |  |  |  |  |  |  |  |  |  |  |  |  |  |  |  |  | 1 |
| GRP94 |  |  |  |  |  |  |  |  |  |  |  |  |  |  |  |  | ○ |  |  |  | 1 |
| GSK3A/B |  | ○ |  |  |  |  |  |  |  |  |  |  |  |  |  |  |  |  |  |  | 1 |
|  |  |  |  |  |  |  |  |  |  |  |  |  |  |  |  |  |  |  |  |  |  |
| **Additional file 3 continue** | | | | | | | | | | | | | | | | | | | | | |
|  | **Ref.** | | | | | | | | | | | | | | | | | | | |  |
| **Protein** | **Buscail, 2019** | **Fahrmann,2020** | **Jin, 2018** | **Kim, 2021** | **Lewis, 2018** | **Li, 2018** | **Li, 2021** | **Liang, 2017** | **Lux, 2019** | **Madhavan, 2015** | **Melo, 2015** | **Moutinho-R, 2021** | **Rodrigues, 2019** | **Verel-Y,2021** | **Wei, 2020** | **Xiao, 2019** | **Yang, 2017** | **Yang, 2021** | **Zheng, 2022** | **Zhou, 2020** | **number of studies** |
| HER2 |  |  |  |  |  |  |  |  |  |  |  |  |  |  |  |  | △ |  |  |  | 1 |
| HIPK3 |  | ○ |  |  |  |  |  |  |  |  |  |  |  |  |  |  |  |  |  |  | 1 |
| HK2 |  | ○ |  |  |  |  |  |  |  |  |  |  |  |  |  |  |  |  |  |  | 1 |
| HSD17B10 | | ○ |  |  |  |  |  |  |  |  |  |  |  |  |  |  |  |  |  |  | 1 |
| IBSP |  | ○ |  |  |  |  |  |  |  |  |  |  |  |  |  |  |  |  |  |  | 1 |
| IDE |  | ○ |  |  |  |  |  |  |  |  |  |  |  |  |  |  |  |  |  |  | 1 |
| IDUA |  | ○ |  |  |  |  |  |  |  |  |  |  |  |  |  |  |  |  |  |  | 1 |
| IFNB1 |  | ○ |  |  |  |  |  |  |  |  |  |  |  |  |  |  |  |  |  |  | 1 |
| IFNG |  | ○ |  |  |  |  |  |  |  |  |  |  |  |  |  |  |  |  |  |  | 1 |
| IFNGR1 |  | ○ |  |  |  |  |  |  |  |  |  |  |  |  |  |  |  |  |  |  | 1 |
| IFNGR2 |  | ○ |  |  |  |  |  |  |  |  |  |  |  |  |  |  |  |  |  |  | 1 |
| IL12RB2 |  | ○ |  |  |  |  |  |  |  |  |  |  |  |  |  |  |  |  |  |  | 1 |
| IL17F |  | ○ |  |  |  |  |  |  |  |  |  |  |  |  |  |  |  |  |  |  | 1 |
| IL17RB |  | ○ |  |  |  |  |  |  |  |  |  |  |  |  |  |  |  |  |  |  | 1 |
| IL1A |  | ○ |  |  |  |  |  |  |  |  |  |  |  |  |  |  |  |  |  |  | 1 |
| IL1RN |  | ○ |  |  |  |  |  |  |  |  |  |  |  |  |  |  |  |  |  |  | 1 |
| IL27RA |  | ○ |  |  |  |  |  |  |  |  |  |  |  |  |  |  |  |  |  |  | 1 |
|  |  |  |  |  |  |  |  |  |  |  |  |  |  |  |  |  |  |  |  |  |  |
| **Additional file 3 continue** | | | | | | | | | | | | | | | | | | | | | |
|  | **Ref.** | | | | | | | | | | | | | | | | | | | |  |
| **Protein** | **Buscail, 2019** | **Fahrmann,2020** | **Jin, 2018** | **Kim, 2021** | **Lewis, 2018** | **Li, 2018** | **Li, 2021** | **Liang, 2017** | **Lux, 2019** | **Madhavan, 2015** | **Melo, 2015** | **Moutinho-R, 2021** | **Rodrigues, 2019** | **Verel-Y,2021** | **Wei, 2020** | **Xiao, 2019** | **Yang, 2017** | **Yang, 2021** | **Zheng, 2022** | **Zhou, 2020** | **number of studies** |
| IL34 |  | ○ |  |  |  |  |  |  |  |  |  |  |  |  |  |  |  |  |  |  | 1 |
| IL36A |  | ○ |  |  |  |  |  |  |  |  |  |  |  |  |  |  |  |  |  |  | 1 |
| IMPDH1 |  | ○ |  |  |  |  |  |  |  |  |  |  |  |  |  |  |  |  |  |  | 1 |
| ING1 |  | ○ |  |  |  |  |  |  |  |  |  |  |  |  |  |  |  |  |  |  | 1 |
| INSR |  | ○ |  |  |  |  |  |  |  |  |  |  |  |  |  |  |  |  |  |  | 1 |
| ISG15 |  | ○ |  |  |  |  |  |  |  |  |  |  |  |  |  |  |  |  |  |  | 1 |
| ITGA2 |  |  |  | △ |  |  |  |  |  |  |  |  |  |  |  |  |  |  |  |  | 1 |
| ITGAV |  |  |  | △ |  |  |  |  |  |  |  |  |  |  |  |  |  |  |  |  | 1 |
| KIF23 |  | ○ |  |  |  |  |  |  |  |  |  |  |  |  |  |  |  |  |  |  | 1 |
| KLK14 |  | ○ |  |  |  |  |  |  |  |  |  |  |  |  |  |  |  |  |  |  | 1 |
| KLK6 |  | ○ |  |  |  |  |  |  |  |  |  |  |  |  |  |  |  |  |  |  | 1 |
| KYNU |  | ○ |  |  |  |  |  |  |  |  |  |  |  |  |  |  |  |  |  |  | 1 |
| LCK |  | ○ |  |  |  |  |  |  |  |  |  |  |  |  |  |  |  |  |  |  | 1 |
| LGALS3BP |  | ○ |  |  |  |  |  |  |  |  |  |  |  |  |  |  |  |  |  |  | 1 |
| LIN7B |  | ○ |  |  |  |  |  |  |  |  |  |  |  |  |  |  |  |  |  |  | 1 |
| LTA LTB |  | ○ |  |  |  |  |  |  |  |  |  |  |  |  |  |  |  |  |  |  | 1 |
| LY9 |  | ○ |  |  |  |  |  |  |  |  |  |  |  |  |  |  |  |  |  |  | 1 |
|  |  |  |  |  |  |  |  |  |  |  |  |  |  |  |  |  |  |  |  |  |  |
| **Additional file 3 continue** | | | | | | | | | | | | | | | | | | | | | |
|  | **Ref.** | | | | | | | | | | | | | | | | | | | |  |
| **Protein** | **Buscail, 2019** | **Fahrmann,2020** | **Jin, 2018** | **Kim, 2021** | **Lewis, 2018** | **Li, 2018** | **Li, 2021** | **Liang, 2017** | **Lux, 2019** | **Madhavan, 2015** | **Melo, 2015** | **Moutinho-R, 2021** | **Rodrigues, 2019** | **Verel-Y,2021** | **Wei, 2020** | **Xiao, 2019** | **Yang, 2017** | **Yang, 2021** | **Zheng, 2022** | **Zhou, 2020** | **number of studies** |
| MAP2K1 |  | ○ |  |  |  |  |  |  |  |  |  |  |  |  |  |  |  |  |  |  | 1 |
| MAPK11 |  | ○ |  |  |  |  |  |  |  |  |  |  |  |  |  |  |  |  |  |  | 1 |
| MAPK12 |  | ○ |  |  |  |  |  |  |  |  |  |  |  |  |  |  |  |  |  |  | 1 |
| MAPK13 |  | ○ |  |  |  |  |  |  |  |  |  |  |  |  |  |  |  |  |  |  | 1 |
| MAPK8 |  | ○ |  |  |  |  |  |  |  |  |  |  |  |  |  |  |  |  |  |  | 1 |
| MAPKAPK2 | | ○ |  |  |  |  |  |  |  |  |  |  |  |  |  |  |  |  |  |  | 1 |
| METAP1 |  | ○ |  |  |  |  |  |  |  |  |  |  |  |  |  |  |  |  |  |  | 1 |
| MIF |  |  |  |  |  | ○ |  |  |  |  |  |  |  |  |  |  |  |  |  |  | 1 |
| MMP13 |  | ○ |  |  |  |  |  |  |  |  |  |  |  |  |  |  |  |  |  |  | 1 |
| MMP14 |  | ○ |  |  |  |  |  |  |  |  |  |  |  |  |  |  |  |  |  |  | 1 |
| MSLN |  | ○ |  |  |  |  |  |  |  |  |  |  |  |  |  |  |  |  |  |  | 1 |
| MUC1 |  |  |  |  |  |  |  |  |  |  |  |  |  |  |  |  | △ |  |  |  | 1 |
| NAPA |  | ○ |  |  |  |  |  |  |  |  |  |  |  |  |  |  |  |  |  |  | 1 |
| NMT1 |  | ○ |  |  |  |  |  |  |  |  |  |  |  |  |  |  |  |  |  |  | 1 |
| PAK7 |  | ○ |  |  |  |  |  |  |  |  |  |  |  |  |  |  |  |  |  |  | 1 |
| PARK7 |  | ○ |  |  |  |  |  |  |  |  |  |  |  |  |  |  |  |  |  |  | 1 |
| PDE9A |  | ○ |  |  |  |  |  |  |  |  |  |  |  |  |  |  |  |  |  |  | 1 |
|  |  |  |  |  |  |  |  |  |  |  |  |  |  |  |  |  |  |  |  |  |  |
| **Additional file 3 continue** | | | | | | | | | | | | | | | | | | | | | |
|  | **Ref.** | | | | | | | | | | | | | | | | | | | |  |
| **Protein** | **Buscail, 2019** | **Fahrmann,2020** | **Jin, 2018** | **Kim, 2021** | **Lewis, 2018** | **Li, 2018** | **Li, 2021** | **Liang, 2017** | **Lux, 2019** | **Madhavan, 2015** | **Melo, 2015** | **Moutinho-R, 2021** | **Rodrigues, 2019** | **Verel-Y,2021** | **Wei, 2020** | **Xiao, 2019** | **Yang, 2017** | **Yang, 2021** | **Zheng, 2022** | **Zhou, 2020** | **number of studies** |
| PDGFRA |  | ○ |  |  |  |  |  |  |  |  |  |  |  |  |  |  |  |  |  |  | 1 |
| PDK1 |  | ○ |  |  |  |  |  |  |  |  |  |  |  |  |  |  |  |  |  |  | 1 |
| PDXK |  | ○ |  |  |  |  |  |  |  |  |  |  |  |  |  |  |  |  |  |  | 1 |
| PDXP |  | ○ |  |  |  |  |  |  |  |  |  |  |  |  |  |  |  |  |  |  | 1 |
| PECAM1 |  | ○ |  |  |  |  |  |  |  |  |  |  |  |  |  |  |  |  |  |  | 1 |
| PIK3CA/R1 | | ○ |  |  |  |  |  |  |  |  |  |  |  |  |  |  |  |  |  |  | 1 |
| PLCG1 |  | ○ |  |  |  |  |  |  |  |  |  |  |  |  |  |  |  |  |  |  | 1 |
| POR |  | ○ |  |  |  |  |  |  |  |  |  |  |  |  |  |  |  |  |  |  | 1 |
| PPP3R1 |  | ○ |  |  |  |  |  |  |  |  |  |  |  |  |  |  |  |  |  |  | 1 |
| PRDX5 |  | ○ |  |  |  |  |  |  |  |  |  |  |  |  |  |  |  |  |  |  | 1 |
| PRKCI |  | ○ |  |  |  |  |  |  |  |  |  |  |  |  |  |  |  |  |  |  | 1 |
| PRLR |  | ○ |  |  |  |  |  |  |  |  |  |  |  |  |  |  |  |  |  |  | 1 |
| RXFP1 |  | ○ |  |  |  |  |  |  |  |  |  |  |  |  |  |  |  |  |  |  | 1 |
| SAA1 |  |  |  |  |  |  |  |  |  |  |  |  |  |  |  |  |  |  | ○ |  | 1 |
| SEMA6A |  | ○ |  |  |  |  |  |  |  |  |  |  |  |  |  |  |  |  |  |  | 1 |
| SEMA6B |  | ○ |  |  |  |  |  |  |  |  |  |  |  |  |  |  |  |  |  |  | 1 |
|  |  |  |  |  |  |  |  |  |  |  |  |  |  |  |  |  |  |  |  |  |  |
|  |  |  |  |  |  |  |  |  |  |  |  |  |  |  |  |  |  |  |  |  |  |
| **Additional file 3 continue** | | | | | | | | | | | | | | | | | | | | | |
|  | **Ref.** | | | | | | | | | | | | | | | | | | | |  |
| **Protein** | **Buscail, 2019** | **Fahrmann,2020** | **Jin, 2018** | **Kim, 2021** | **Lewis, 2018** | **Li, 2018** | **Li, 2021** | **Liang, 2017** | **Lux, 2019** | **Madhavan, 2015** | **Melo, 2015** | **Moutinho-R, 2021** | **Rodrigues, 2019** | **Verel-Y,2021** | **Wei, 2020** | **Xiao, 2019** | **Yang, 2017** | **Yang, 2021** | **Zheng, 2022** | **Zhou, 2020** | **number of studies** |
| SLAMF6 |  | ○ |  |  |  |  |  |  |  |  |  |  |  |  |  |  |  |  |  |  | 1 |
| SPARCL1 |  | ○ |  |  |  |  |  |  |  |  |  |  |  |  |  |  |  |  |  |  | 1 |
| SPHK2 |  | ○ |  |  |  |  |  |  |  |  |  |  |  |  |  |  |  |  |  |  | 1 |
| SSRP1 |  | ○ |  |  |  |  |  |  |  |  |  |  |  |  |  |  |  |  |  |  | 1 |
| STAT6 |  | ○ |  |  |  |  |  |  |  |  |  |  |  |  |  |  |  |  |  |  | 1 |
| STK16 |  | ○ |  |  |  |  |  |  |  |  |  |  |  |  |  |  |  |  |  |  | 1 |
| TGFBR2 |  | ○ |  |  |  |  |  |  |  |  |  |  |  |  |  |  |  |  |  |  | 1 |
| TLR4 |  | ○ |  |  |  |  |  |  |  |  |  |  |  |  |  |  |  |  |  |  | 1 |
| TNF |  | ○ |  |  |  |  |  |  |  |  |  |  |  |  |  |  |  |  |  |  | 1 |
| TNFRSF13B | | ○ |  |  |  |  |  |  |  |  |  |  |  |  |  |  |  |  |  |  | 1 |
| TNFRSF14 |  | ○ |  |  |  |  |  |  |  |  |  |  |  |  |  |  |  |  |  |  | 1 |
| TNFRSF18 |  | ○ |  |  |  |  |  |  |  |  |  |  |  |  |  |  |  |  |  |  | 1 |
| TNFSF11 |  | ○ |  |  |  |  |  |  |  |  |  |  |  |  |  |  |  |  |  |  | 1 |
| TNFSF13B |  | ○ |  |  |  |  |  |  |  |  |  |  |  |  |  |  |  |  |  |  | 1 |
| TNFSF14 |  | ○ |  |  |  |  |  |  |  |  |  |  |  |  |  |  |  |  |  |  | 1 |
| TOP1 |  | ○ |  |  |  |  |  |  |  |  |  |  |  |  |  |  |  |  |  |  | 1 |
|  |  |  |  |  |  |  |  |  |  |  |  |  |  |  |  |  |  |  |  |  |  |
|  |  |  |  |  |  |  |  |  |  |  |  |  |  |  |  |  |  |  |  |  |  |
| **Additional file 3 continue** | | | | | | | | | | | | | | | | | | | | | |
|  | **Ref.** | | | | | | | | | | | | | | | | | | | |  |
| **Protein** | **Buscail, 2019** | **Fahrmann,2020** | **Jin, 2018** | **Kim, 2021** | **Lewis, 2018** | **Li, 2018** | **Li, 2021** | **Liang, 2017** | **Lux, 2019** | **Madhavan, 2015** | **Melo, 2015** | **Moutinho-R, 2021** | **Rodrigues, 2019** | **Verel-Y,2021** | **Wei, 2020** | **Xiao, 2019** | **Yang, 2017** | **Yang, 2021** | **Zheng, 2022** | **Zhou, 2020** | **number of studies** |
| TPSG1 |  | ○ |  |  |  |  |  |  |  |  |  |  |  |  |  |  |  |  |  |  | 1 |
| Tspan8 |  |  |  |  |  |  |  |  |  | △ |  |  |  |  |  |  |  |  |  |  | 1 |
| TYRO3 |  | ○ |  |  |  |  |  |  |  |  |  |  |  |  |  |  |  |  |  |  | 1 |
| UBC |  | ○ |  |  |  |  |  |  |  |  |  |  |  |  |  |  |  |  |  |  | 1 |
| UBE2G2 |  | ○ |  |  |  |  |  |  |  |  |  |  |  |  |  |  |  |  |  |  | 1 |
| VEGFA |  | ○ |  |  |  |  |  |  |  |  |  |  |  |  |  |  |  |  |  |  | 1 |
| WNT2 |  |  |  |  |  |  |  |  |  |  |  |  |  |  |  |  | △ |  |  |  | 1 |
| YES1 |  | ○ |  |  |  |  |  |  |  |  |  |  |  |  |  |  |  |  |  |  | 1 |
| ZAP70 |  | ○ |  |  |  |  |  |  |  |  |  |  |  |  |  |  |  |  |  |  | 1 |
| ZIP4 |  |  | ○ |  |  |  |  |  |  |  |  |  |  |  |  |  |  |  |  |  | 1 |

○ represents proteins which have only been analyzed individually and not as part of a protein panel; △ represents proteins which are part of a panel.
