# Supplementary figures and images for: Extracellular vesicle biomarkers for pancreatic cancer diagnosis: a systematic review and meta-analysis
Source: BMC Cancer. 2022 May 23;22:573. doi: 10.1186/s12885-022-09463-x (PMC9125932; doi:10.1186/s12885-022-09463-x)

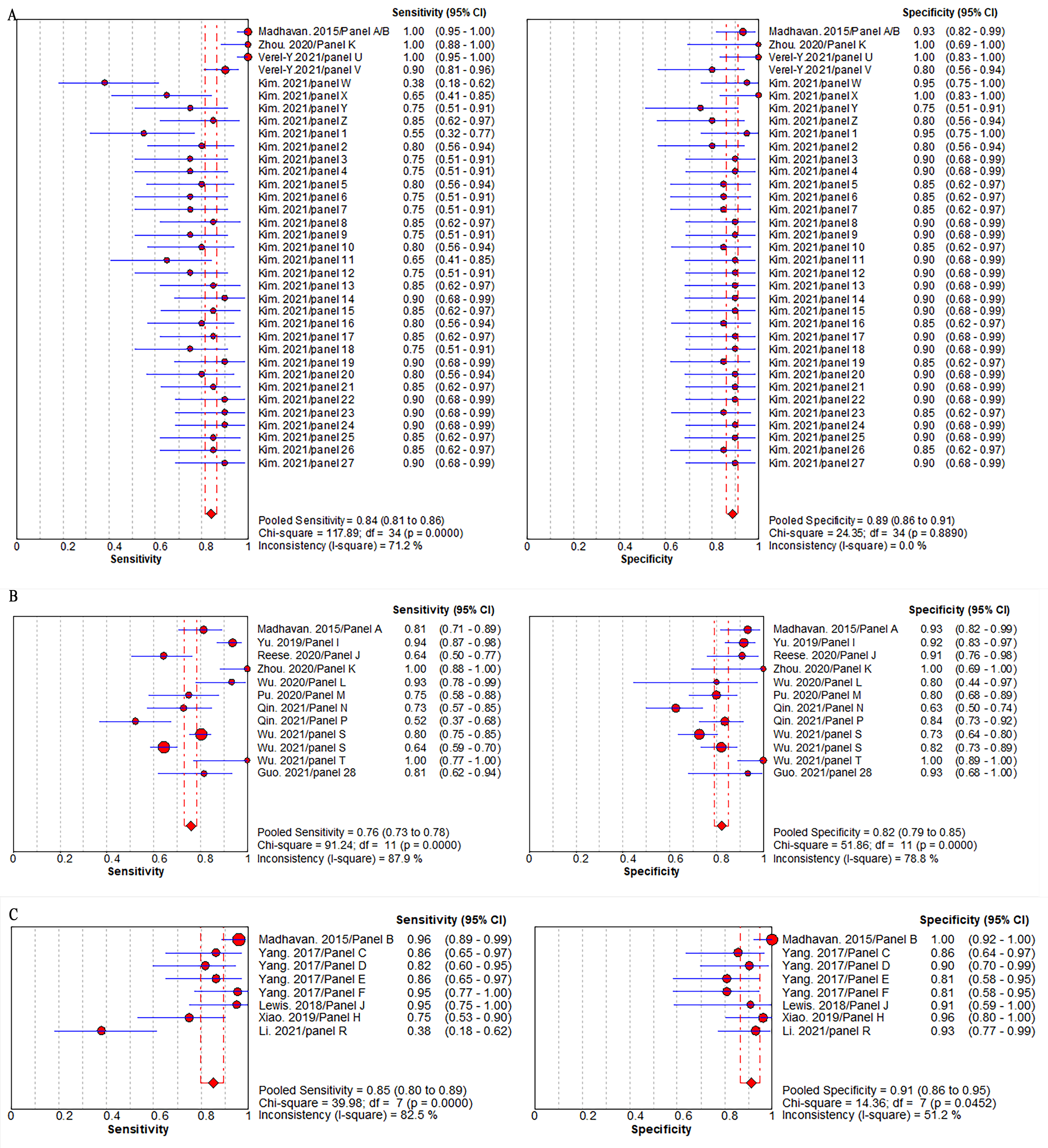

Supplement: Supplementary file 4 — Additional file 4. Pooled sensitivity and specificity of EV biomarker panels for pancreatic cancer diagnosis. [file 12885_2022_9463_MOESM4_ESM.tif]
